# Effect of the spatial form of outpatient buildings on energy consumption in different climate zones in China

**DOI:** 10.1371/journal.pone.0293982

**Published:** 2023-11-09

**Authors:** Jiao Yang, Qun Zhang

**Affiliations:** School of Architecture, Xi’an University of Architecture and Technology, Xi’an, Shanxi, China; Central Queensland University, AUSTRALIA

## Abstract

Under the influence of global epidemics and the need for urban expansion, many outpatient buildings have been rapidly constructed, but the problem of high energy consumption has been neglected. There is a lack of research on the impact of outpatient building forms on energy consumption in different climate zones. Many studies have demonstrated that the energy consumption of a given building can be greatly reduced by adopting a reasonable spatial form design at the early stages of design. Therefore, if architects choose a reasonable spatial form, this could effectively reduce energy consumption. In this study, outpatient building cases in China were summarized, and three typical spatial forms were proposed: the centralized, corridor, and courtyard forms. The DesignBuilder tool was used to simulate and analyse the typical building energy consumption in different climate zones. The results showed that the corridor form (southwards) should be chosen in the severe cold zone, the centralized form (southwards) should be chosen in the cold zone and the hot summer and cold winter zone, the centralized form (northwards) should be chosen in the hot summer and warm winter zone, and the centralized or corridor form can be chosen in the warm zone. The results of this study could provide a reference for energy-efficient design of outpatient buildings in China and other regions with similar conditions and could help architects quickly select reasonable spatial forms at the early stages of design.

## 1. Introduction

**Fig 1:**
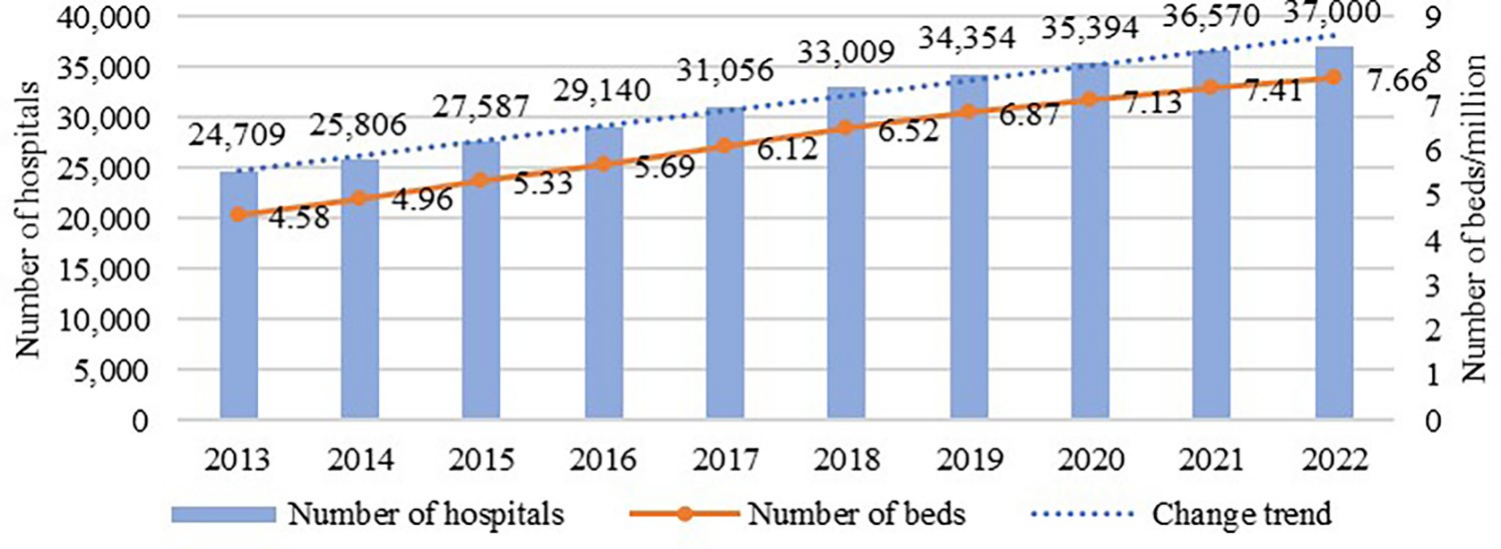
Changes in the number and size of hospital buildings in China, 2013–2022.

Over the last decade, with the continuous development of healthcare services in China, the number and scale of hospital buildings have rapidly increased (Fig 1). According to the China Bureau of Statistics [1], in 2022, the number of hospitals increased from 24,709 to 37,000, approximately 1.5 times that in 2013, and the total number of hospital beds increased from 4.58 million to 7.66 million, approximately 1.7 times that in 2013. However, the energy consumption of hospital buildings also shows a rising trend, approximately 5–10% per year [2]. Energy efficiency is particularly critical in hospital buildings. Hospitals are unique buildings that are operated 24 hours a day and provide multifunctional services, and their energy consumption is higher than that of general public buildings due to the large number of people, high frequency of use, and high population density [3]. The energy consumption of hospital buildings in China is 1.6–2 times higher than that of general public buildings and more than twice that of the same types of hospital buildings in developed countries [4]. In terms of the energy consumption distribution, out- and inpatient buildings account for 30–70% and 25–40%, respectively, of the total energy consumption [5]. Heating and cooling energy consumption accounts for the largest proportion of energy consumption in outpatient buildings [6–9]. Therefore, energy-efficient design of hospital outpatient buildings is very important.

One of the main parameters that can have a significant impact on the energy consumption of a building is the building form. Building form determines how a building interacts with its environment, and it can increase or decrease exposure to direct sunlight, humidity, rain, prevailing winds and shadows, and self-shading of the building. Hemsath and Alagheband Bandhosseini **[10]**, by comparing geometric variations and material factors in energy consumption, stated that building form and orientation are early decisions in the design process that can significantly impact energy consumption, lighting, cooling, and heating loads. Pacheco et al **[11]** in reviewing the literature on sustainable building design concluded that the factors that have the greatest impact on the final energy demand are the building orientation, shape, and the ratio between the building’s external surfaces and the building volume. Of these factors, modifying building form and orientation after building construction is extremely challenging. Therefore, the influence of the form and orientation of outpatient buildings on energy performance is very important and deserves to be studied in depth.

The following is a brief review of relevant studies involving the impact of spatial form on energy consumption under different climatic conditions. In Ref. **[12]**, a study on the building layout pattern and energy efficiency of hospitals in cold climate zones in China found that the energy-saving rate of the outpatient building with a grid courtyard form was the highest (16.3%).In Ref. **[13]**, based on the climate of the Guangzhou area, the light and ventilation energy consumption of outpatient buildings with different layout forms of outpatient buildings were tested for light and ventilation energy consumption, and the results showed that outpatient buildings with open courtyards consume less energy compared to closed courtyards. In Ref. **[14]**, the energy consumption of buildings was analysed through measured data in a general hospital in a hot summer and cold winter zone, and it was found that air-conditioning energy consumption accounted for 40% of the total energy consumption for the whole year. In Ref. **[15]**, the optimal form of rectangular plan buildings in hot zones can reduce direct solar radiation by up to 20%.In Ref. **[16]**, the optimal building form to reduce cooling loads was investigated using the computer simulation programme Autodesk Ecotect and it was found that dispersed building forms have higher cooling loads compared to compact building forms. In Ref. **[17]**, the effect of building form variables (e.g., window-to-wall ratio, orientation, and number of floors) on energy consumption in cold climates was investigated, and it was found that the number of floors had the greatest effect on heating energy, while the overall size of the building had the greatest effect on cooling and electricity use. In Ref. [18], to determine the optimal building form and orientation, the energy consumption of six different building forms for one year was simulated based on the climatic characteristics of the city of Kirkuk, Iraq, and it was found that the T-shape model had the lowest energy consumption at a rotation angle of 285°. In Ref. **[19]**, rectangular buildings with plan aspect ratios between 1:1.25 and 1:1.5 consumed less energy than other forms (with 1:1 plan aspect ratios) when longer spans were oriented towards the north in a specific area of Kuwait. In Ref. **[20]**, a comprehensive analysis of the important parameters (thermal transmittance, glazing size and its orientation, aspect ratio, horizontal and vertical extension) of 216 box log house models from both Athens and Seville was carried out to identify the parameters that have a significant impact on the energy consumption. In Ref. **[21]**, it was investigated to minimise insolation while retaining the total floor area and optimising the building form could reduce insolation by up to 48%. In Ref. **[22]**, a new design workflow methodology was proposed to comprehensively explore performance-based design options at the building scale. Based on these studies, it is relevant to fully investigate the impact of spatial form on energy consumption in outpatient buildings.

However, most of the related studies changed some of the other parameters when considering the spatial form variables of the building. For example, in addition to spatial morphology **[13],** changed the window-to-wall ratio and functional layout of the model; and **[17]** changed the building floors. Mixing spatial form parameters with other design parameters makes it impossible to judge the isolated effect of spatial form on energy consumption. In addition, most of the related studies were derived from analyses of building energy consumption under the same climatic conditions **[12–14, 18]**. China is a vast country with large differences in climatic and environmental conditions, which can be divided into five climatic zones. So far, the impact of spatial forms of outpatient buildings on energy consumption under various climatic conditions has not been quantified, and there is a lack of comparative studies on energy consumption under different spatial forms in different climatic zones. A systematic study of the effect of outpatient building spatial form on energy consumption is needed, in which the other parameters of the model are held constant to determine the separate effects of spatial form. This study investigated the effect of space form on heating and cooling energy consumption using three typical outpatient space forms as references and compared energy consumption in five climate zones. Recommendations are provided for the selection of outpatient building space forms for different climate zones at the design stage.

## 2. Materials and methods

**Fig 2:**
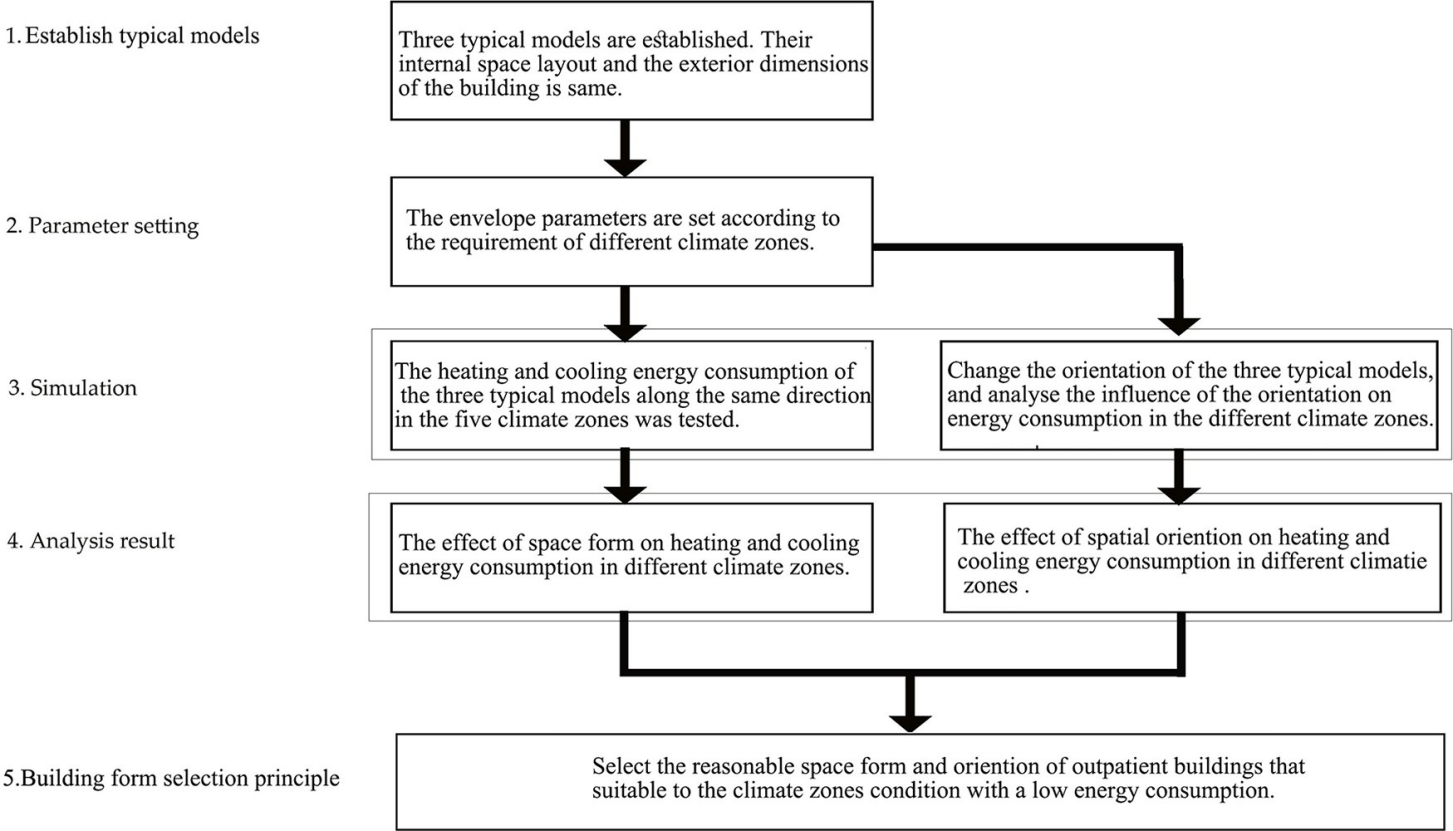
Building form simulation procedure.

The research methodology and steps for the study of the impact of common outpatient building forms on energy consumption in the different climate zones are shown in Fig 2. First, the outpatient building forms were classified, and a typical model for energy consumption simulation was established based on the literature and case studies. Second, suitable simulation software was selected, various parameters were set, and the study object and target (heating and cooling energy consumption) were defined. Finally, the simulation results were analysed to define a reasonable spatial form for adoption in the different climate zones. Key issues throughout the analysis process included the construction of the typical model and the setting of simulation parameters.

### 2.1. Establishing the typical model

**Fig 3:**
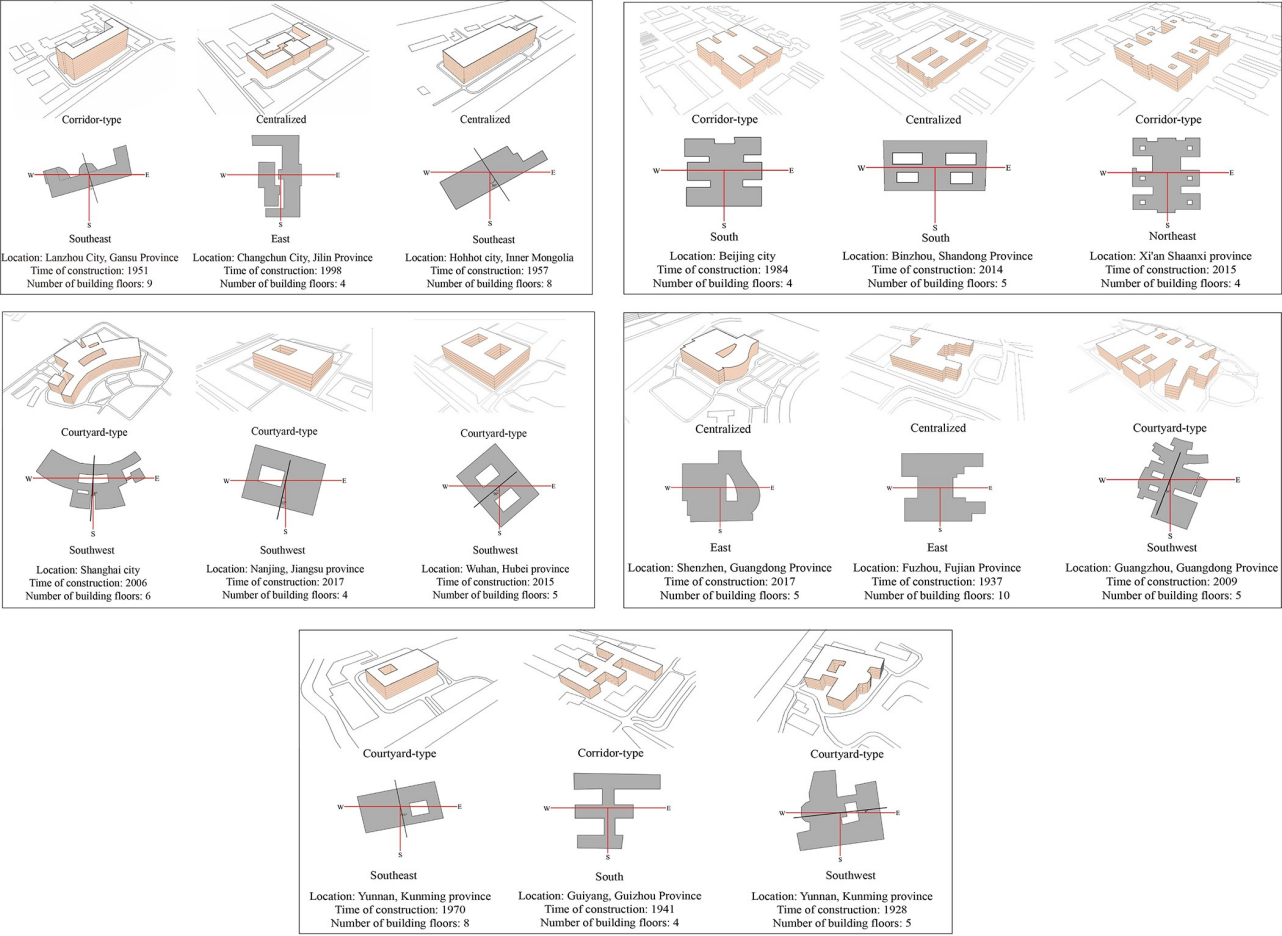
Case studies of the outpatient building forms and orientations in the different climate zones in China. (a) Severe cold zone. (b) Cold zone. (c) Hot summer and cold winter zone. (d) Hot summer and warm winter zone. (e) Warm zone.

To better understand the spatial form characteristics of outpatient buildings, relevant research literature was reviewed (Table 1), with a summary of the study timing, research focus, and content. The literature review revealed that due to the specific functional characteristics of outpatient buildings, their spatial forms exhibit regularity. The spatial form of early outpatient buildings was scattered and chaotic. This phenomenon may be due to the limitations of the building sites, which necessitated extensions and energy-saving renovations. However, this could also indicate that the physical form of these buildings was not designed with energy efficiency in mind. Therefore, these cases are not representative. In recent years, a certain similarity among buildings and a clear classification of building forms have occurred. Based on the relationship between the building blocks, buildings can be broadly classified as centralized, corridor-type, courtyard-type, and slab buildings. In addition, to better understand the spatial forms of the general outpatient buildings in China in the different climate zones, relevant case information, including the building form, orientation, and regional location, was examined in this paper (Fig 3). As shown in Table 2, the sample building forms differed in the different climate zones, with the main types including centralized, corridor-type, and courtyard-type buildings. Most sample buildings exhibited a positive orientation (east-west or north-south). These building forms and orientations provided the raw data and basic conditions for establishing typical models, ensuring reasonable conclusions.

**Table 1:** Review of outpatient building research.

Scholar	Country	Time	Research Direction	Types of Forms Mentioned/Analysed
Huang Xiqiu et al. [23]	China	2001	Building density	Centralized, semi-centralized, and decentralized.
Ma Jin et al. [24]	China	2009	Plane layout	Corridor-type, courtyard-type, and combinations.
Zhang Shizhi et al. [25]	China	2015	Plane layout	Enclosure, courtyard-type, street, and centralized.
Ines Verena Arnolds et al. [26, 27]	Germany	2017	Pathway design	Courtyard-type, and semi-centralized.
Sun Bing et al. [28]	China	2018	Plane layout	Corridor-type, hall, and plate.
Yang Jun et al. [29]	China	2019	Plane layout	Hall, joint hall, and street.
Wu Shaopeng et al. [30]	China	2019	Building density	Street, courtyard-type, and plate.
Joshua Caplan et al. [27]	UK	2020	Social distance	Centralized, semi-centralized, street, and decentralized.
Sri Hartuti Wahyuningrum et al. [31]	Indonesia	2020	Space utilization efficiency	Decentralized and centralized.
Zhaochao Wang et al. [32]	China	2021	Pathway design	Centralized and corridor-type.

**Table 2:** Five typical cities in the five climate zones in this study.

Climate zones	Typical city
Severe cold	Harbin
Cold	Xi’an
Hot summer and cold winter	Shanghai
Hot summer and cold winter	Shenzhen
Warm	Kunming

**Fig 4:**
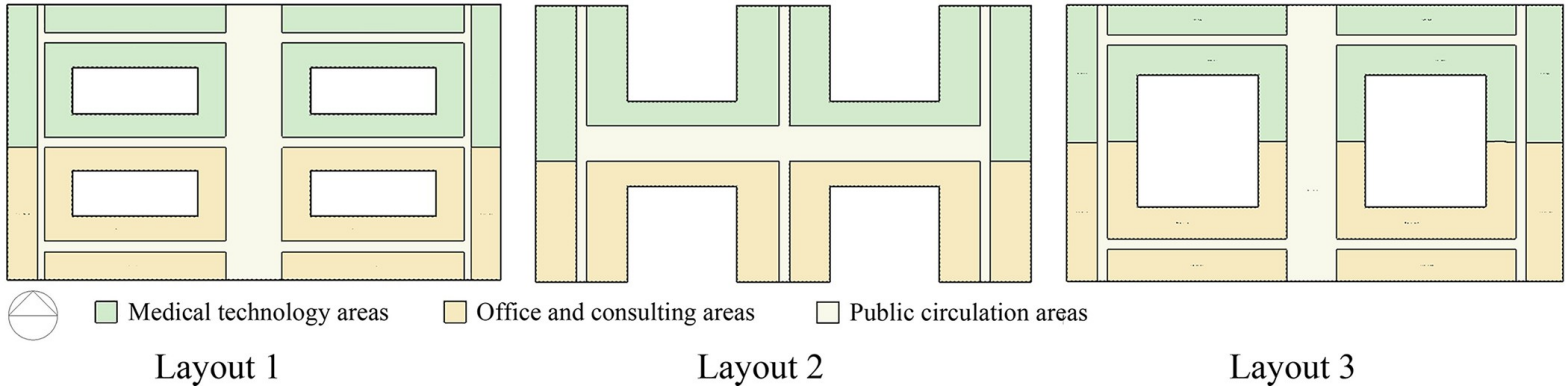
Layout of the three typical models: (a) Centralized form; (b) corridor form; and (c) courtyard form.

Typical models were developed according to the following principles to yield comparable results. Combining the results of the literature review in Table 1, the samples in Table 2 were screened to eliminate older, invalid samples. The form of the typical model was simplified to increase its representativeness. The spatial form of the typical model should meet the functional requirements of outpatient buildings, including three parts: medical technology area, office area, and public transport area. To ensure that the simulation results of the three typical models are comparable, their layouts should be consistent. Based on the above principles, typical models were established. The specific information of the three typical models is as follows: (1) centralized form: the building form closely resembles a square, with no or very small openings and a small contact area with air (Fig 4A). (2) Corridor form: a main corridor connects the different functional areas, with open outdoor spaces between the various functional areas and a large surface area in contact with air (Fig 4B). (3) Courtyard form: one or two large outdoor spaces occur inside the building. All the rooms are arranged around the courtyard, exhibiting a moderate contact area with the outside air (Fig 4C).

**Fig 5:**
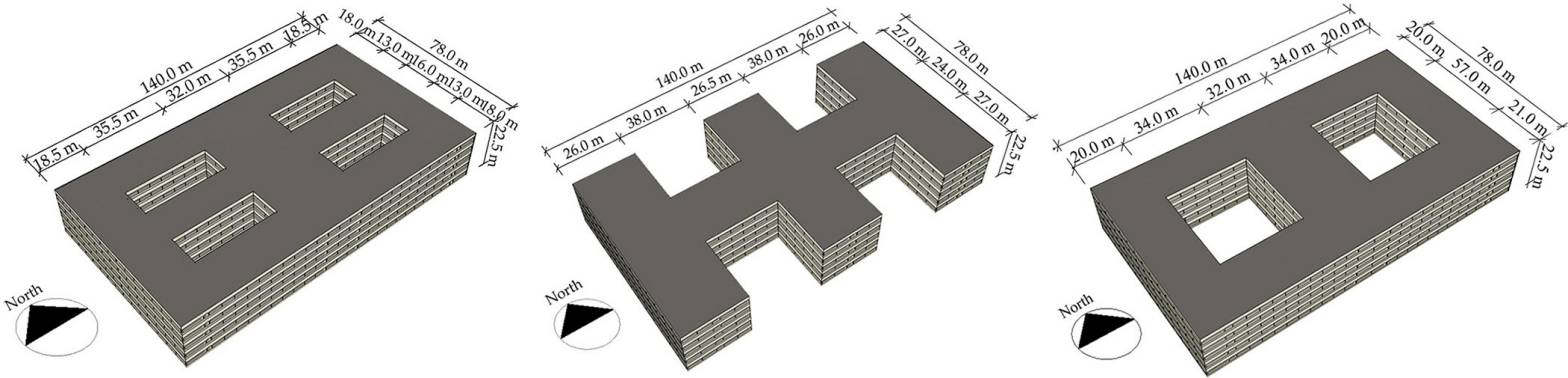
Basic information of the three typical models: (a) Centralized form; (b) corridor form; and (c) courtyard form.

This study focused on the relationship between changes in the building form and energy consumption in the different climate zones. Many factors influence the energy consumption of buildings. To ensure the accuracy of the simulation results under the influence of the single factor of the building form, the typical models should follow the following principles: (1) parameters such as the form factor, window-to-wall ratio, and external boundaries of the model should remain constant within the same climate zone environment. (2) Based on the survey results, the typical models should be consistent with the predominant types of outpatient buildings in most regions. (3) Combined with the sampling survey results, the external profiles of the three models were set as rectangles with the same basic information (Fig 5) (external profile dimensions: 140.0 m × 78.0 m; floor height: 5.5 m; number of floors: 5; form factor: 0.3; window-to-wall ratio: 0.4).

### 2.2. Simulation environment setting

China’s climate greatly varies from region to region due to its vast territory, varying latitudes, and different environmental conditions. Different climatic conditions can lead to distinct energy conservation requirements for buildings. From the perspective of building thermal design, the Thermal Design Code for Civil Buildings (GB50176-2016) divides the Chinese building climate into five zones [33]. In this study, typical cities in the five climate zones were selected for energy consumption simulation (Table 2).

Harbin is located in the severe cold zone, with long cold winters (average temperature of the coldest month -15.8°C), short cool summers (average temperature of the hottest month 23.6°C), large solar radiation, and abundant sunshine. The energy-saving design of buildings in this climate zone focuses on cold protection and heat preservation and does not consider heat protection in summer. Xi’an is located in the cold zone, with long and cold dry winters (average temperature of the coldest month -1. 2~ 0. 0°C), hot and humid summers (average temperature of the hottest month 26. 3~ 26. 6°C), a large annual difference in temperature and abundant sunshine. The energy-saving design of buildings in this region focuses on thermal insulation in winter, ventilation, and shading in summer. Shanghai is located in the hot summer and cold winter area, with a sultry summer, humid and cold winter, small daily difference in temperature, and little sunshine. The energy-saving design of buildings in this region focuses on heat protection and ventilation in summer and cold protection in winter. Shenzhen is located in the hot summer and warm winter area, with a long summer and mild climate, with an annual average temperature of 23.3°C. The energy-saving design of buildings in this area needs to give full consideration to heat prevention and ventilation in summer, and no consideration to heat preservation in winter. Kunming is located in the mild zone, and most of the area is warm in winter and cool in summer, with small difference in temperature throughout the year and less sunshine. The energy-saving design of buildings in this region needs to consider ventilation and moisture protection, without considering heat protection. Overall, these five cities have a wide range of climates, all with typical characteristics of each climate zone, and all have great potential for energy-efficient design. Shanghai and Shenzhen are affected by seasonal typhoons, but most of the year-round climate is still similar to the characteristics of the climate zones.

### 2.3. Simulation tool and parameter settings

In this study, the simulation tool DesignBuilder was used to dynamically simulate the energy consumption of hospital outpatient buildings. This tool is a dynamic simulation and analysis program to obtain the total energy consumption of a building for cooling, heating, lighting, and ventilation. The effectiveness of the DesignBuilder tool in building energy consumption simulation has been confirmed in several studies [34, 35]. The DesignBuilder tool provides an authoritative database of meteorological parameters, including specific design meteorological parameters and simulated winter and summer meteorological parameters. The energy consumption for heating and cooling was simulated for each of the typical models in the selected cities in the different climate zones.

Appropriate parameter settings are a prerequisite for ensuring the validity and accuracy of the simulation results. The selection of the DesignBuilder function panel should be targeted according to the simulation objectives. This study focused on cooling and heating energy consumption. Therefore, the main parameters to be considered include human activity, building envelope, and air conditioning system use. Hospital building parameter templates are predefined in DesignBuilder and include various staff activity arrangements, winter, and summer temperature settings, lighting criteria, air conditioning operation schedules, fresh air volume criteria, etc. Other parameters about the building structure are set regarding the Design Standard for Energy Efficiency of PublicBuildings (GB50189-2015).

#### 2.3.1. Meteorological parameters

The primary climate data for this study was obtained from EnergyPlus [36], using the China Standard Weather Data (CSWD) data source. Other climate-related data were obtained from the Thermal Design Code for Civil Buildings (GB50176-2016), as shown in Table 3. The climate varies greatly from city to city, so it is necessary to design outpatient building forms that match the climatic conditions to reduce energy consumption.

**Table 3:** Meteorological parameters of five selected cities.

		Harbin	Xi’an	Shanghai	Shenzhen	Kunming
Summer						
Mean solar irradiance(W/㎡) (July)	S	75.4	93.5	76.4	58.9	61.2
	W(E)	31.9	151.8	151.6	145.1	148.3
	N	25.3	72.0	67.4	81.1	86.0
	H	145.8	312.0	315.4	304.9	310.9
Mean wind speed (m/s)		3.2	1.7	3.8	3.2	2.1
The mean temperature of the hottest month(°C)		23.8	27.8	28.5	28.4	20.3
Annual cooling degree-days(base 26°C)		14	153	119	374	0
Winter						
Mean solar irradiance(W/㎡) (January))	S	128.9	104.4	136.2	215.8	193.3
	W€	49.7	59.2	63.7	73.8	84.5
	N	28.0	42.8	53.1	40.3	46.6
	H	697	91.4	103.9	183.7	128.2
Mean wind speed (m/s)		3.6	1.7	3.0	2.2	2.5
The mean temperature of coldest month(°C)		16.9	0.9	3.5	4.9	9.4
Annual heating degree-days(base 18°C)		5032	2178	1540	223	1103

#### 2.3.2. Personnel activity parameters

**Fig 6:**
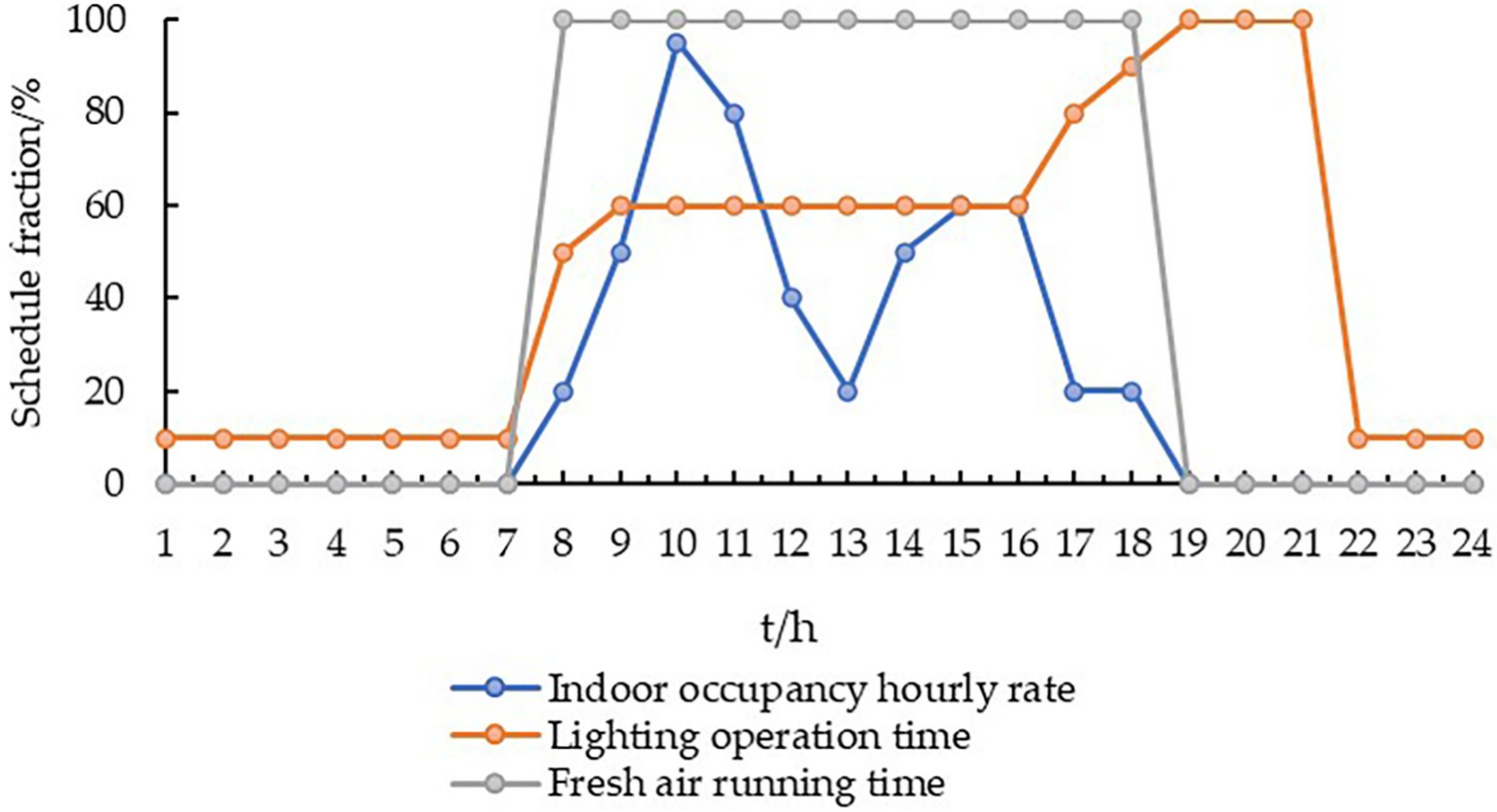
Operation time of the outpatient building system [38].

Human activity in outpatient buildings significantly impacts the resultant energy consumption. Therefore, this parameter should be established according to Chinese design standards and the actual hospital conditions [37] (Table 4). The operating hours and characteristics of general outpatient buildings greatly influence their combined energy consumption patterns. Outpatient clinic operating hours typically extend from 8:30–17:30, with a break at noon (Fig 6) [38]. In general, the number of patients in the morning reaches a peak from 09:00–10:00 and approaches the average at 11:00 [39]. The afternoon peak persists from 14:30–16:00 and then gradually decreases. To ensure the comparability of the results, the same schedule and room design parameters were assumed in the three typical models.

**Table 4:** Room design parameters.

Function	Temperature (summer/winter)	Humidity (summer/winter)	Occupant density (persons/m^2^)	Power density of equipment (W/m^2^)
Outpatient department	26°C/20°C	60%/40%	0.17	20
Medical technology department	26°C/22°C	65%/30%	0.10	64
Circulation space	27°C/18°C	60%/35%	0.25	20

#### 2.3.3. Envelope structure parameters

Building energy consumption is closely related to the heat transfer coefficient, thermal resistance value, building material characteristics, material thickness, and other factors of the building envelope. Therefore, to ensure the accuracy of the simulation results, in this study, the envelope parameters were set according to the ***Code for Thermal Design of Civil Buildings*** in the different climate zones [33]. The specific parameters are provided in the tables below (Tables 5–7).

**Table 5:** Details of the glazing used in the layout models in the five climate zones.

Details of the glazing used	Harbin	Xian	Shanghai	Shenzhen	Kunming
U value (W/m^2^·K)	1.93	2.32	2.58	2.61	2.85
Solar heat gain coefficient (SHGC)	0.63	0.63	0.35	0.30	0.36

**Table 6:** Details of the walls considered in the layout models in the five climate zones.

	Construction of the exterior wall	
Location	Layers (from inside to outside)	U value (W/m^2^·K)
Harbin	20 mm gypsum plaster+120 mm concrete blocks (medium-weight (MW))+70 mm foam-polyurethane, freon-filled+120 mm concrete blocks (lightweight)	0.29
Xian	20 mm gypsum plaster+200 mm concrete blocks (lightweight)+50 mm foam—polyurethane+20 mm lime plaster	0.32
Shanghai	20 mm gypsum plaster+200 mm concrete blocks (MW)+35 mm foam-polyisocyanate+20 mm lime plaster	0. 55
Shenzhen	20 mm gypsum plaster+200 mm concrete blocks (lightweight)+20 mm lime plaster	0.77
Kunming	25 mm cement plaster+200 mm hollow concrete blocks (lightweight)+20 mm gypsum plaster	1.48
**Construction of the interior wall**		
	20 mm gypsum plaster+180 mm concrete blocks (MW)+20 mm gypsum plaster	1.4

**Table 7:** Details of the floors and roofs considered in the layout forms in the five climate zones.

	Construction of the roof	
Location	Layers (from inside to outside)	U value (W/m^2^·K)
Harbin	20 mm gypsum plaster+120 mm cast concrete (lightweight)+60 mm air gap (downwards)+90 mm foam-polyurethane, freon-filled+10 mm asphalt	0.26
Xian	20 mm gypsum plaster+100 mm cast concrete (lightweight)+50 mm air gap (downwards)+60 mm foam-polyurethane+10 mm asphalt	0.35
Shanghai	20 mm gypsum plaster+100 mm cast concrete (lightweight)+50 mm air gap (downwards)+40 mm foam-polyurethane+10 mm asphalt	0.47
Shenzhen	20 mm gypsum plaster+100 mm cast concrete (lightweight)+50 mm air gap (downwards)+25 mm MW glass wool (rolls)+10 mm asphalt	0.76
Kunming	25 mm gypsum plaster+100 mm cast concrete (lightweight)+50 mm air gap (downwards)+25 mm MW glass wool (rolls)+10 mm asphalt	0.76
	**Construction of the floor**	
	9 mm ceramic floor tiles+20 mm cement screed+100 mm cast concrete (lightweight)+20 mm plaster ceiling tiles	1.63

#### 2.3.4. Air conditioning system parameters

Regarding heating, ventilation, and air conditioning systems in hospital buildings, the calculation time of heating energy consumption was consistent with the heating period in the typical cities in each climate zone. The heating period in Harbin lasts from October to April of the following year, the heating period in Xi’an ranges from November to March of the following year, and the heating period in Shanghai extends from December to February of the following next year. The cooling period was automatically initiated at temperatures higher than 30°C. The air conditioning system is a multiline + fresh air system. The fresh air system was set according to the fresh air requirements of the different functional areas. The running time is determined by the DesignBuilder tool based on the calculated indoor cooling and heating temperatures and the set timetable. According to actual hospital conditions, the coefficient of performance (COP) of the multiline cooling system was set to 3.0 and that of the heating system was set to 2.5.

### 2.4. Data validation

**Fig 7:**
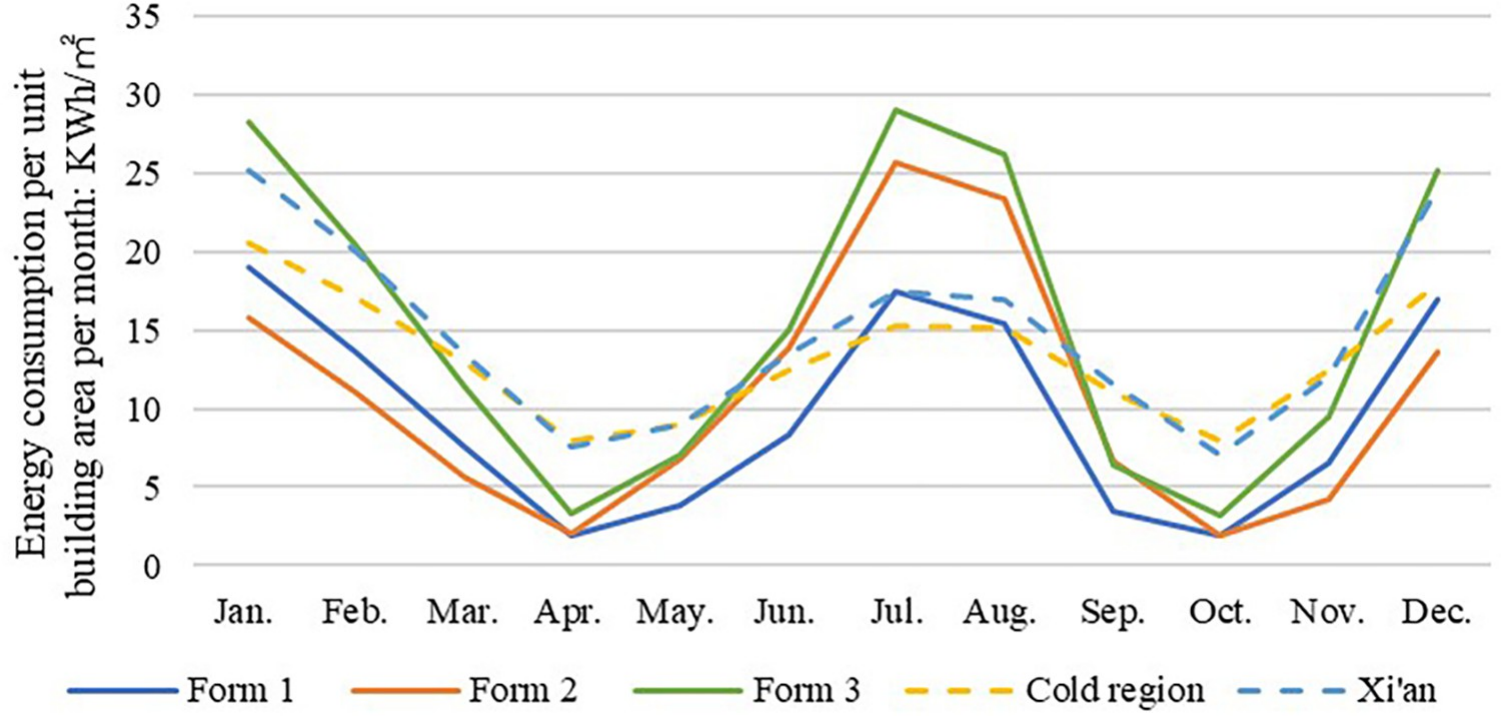
Comparison of the simulated energy consumption of the three typical models with the average energy consumption in the cold zone.

To validate the output data from the DesignBuilder software and to ensure the appropriateness of the problem solving approach, a similar study was selected in which the energy consumption of 30 public hospitals in a cold region of China (including Xi’an) was investigated [12]. In this study, the simulated energy consumption data of three typical models in the cold region (Xi’an) for each month of the year were compared with the results of this survey (Fig 7), which showed that the simulated data showed the same trend as the survey data in [12], and the differences between the data were within a reasonable range. Therefore, the data and problem-solving methods in this study are valid.

## 3. Results

### 3.1. Analysis of the effect of the building form on energy consumption

In this study, the dynamic annual energy consumption was simulated with the three typical models. The simulation results were transformed into energy-saving rates for analysis. The energy-saving rate is a comprehensive indicator reflecting the degree of energy-saving [40], and the energy-saving rate can be calculated with Eq (1). In the energy-saving rate calculation based on the different forms, the average energy consumption of all forms was used as the reference value for the building energy consumption. Cooling and heating energy consumption values were included in the energy consumption per unit of the floor area.


$$R=1-E_{1}\times (1-r)/E_{2}$$ (1)

*R—*Energy-saving rate;

*E*1—Building energy consumption value;

*r*—Energy-saving standard percentage, at r = 75% (General Code for Energy Efficiency and Renewable Energy Application in Buildings—GB 55015–2021); and

*E*_2_—Reference building energy consumption value.

#### 3.1.1. Total energy consumption analysis

**Fig 8:**
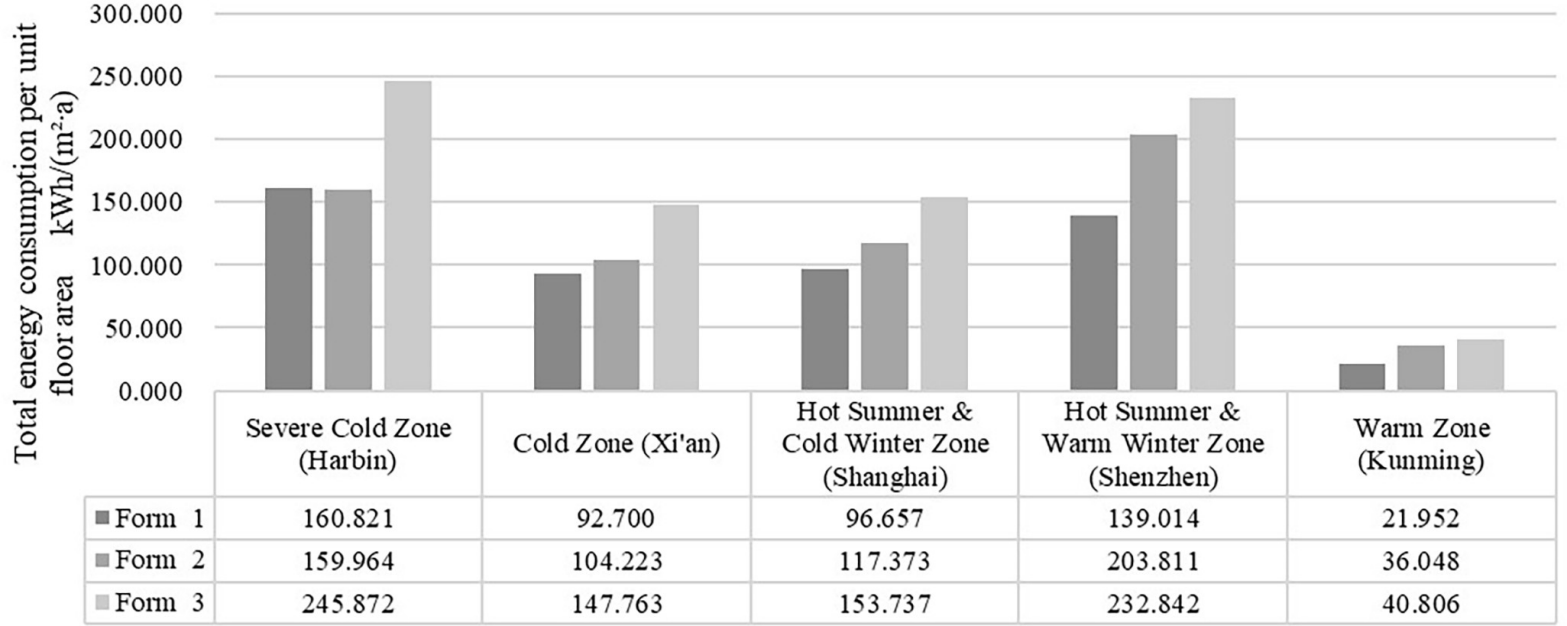
Total heating and cooling energy consumption per unit floor area.

The total energy consumption determined in this section refers to the total energy consumption for heating and cooling. The energy consumption was simulated considering the different building forms, and the results were divided into five groups by climate zone. As shown in Fig 8, the order of the total energy consumption remained the same for each group: Form 3 > Form 2 > Form 1. Comparing the five groups of simulation results, the warm climate zone attained the lowest combined energy consumption, while the severe cold climate zone and the hot summer and cold winter climate zone attained relatively high total energy consumption levels, with that in the latter zone approximately 4–8 times higher than that in the former zone. This result suggests that heating and cooling energy consumption accounts for a large proportion of the building energy consumption. The results for Forms 1 and 2 did not differ much in the severe cold zone, and both could be considered reasonable choices, while Form 1 was the best choice in the other climate zones.

#### 3.1.2. Heating and cooling energy consumption analysis

**Fig 9:**
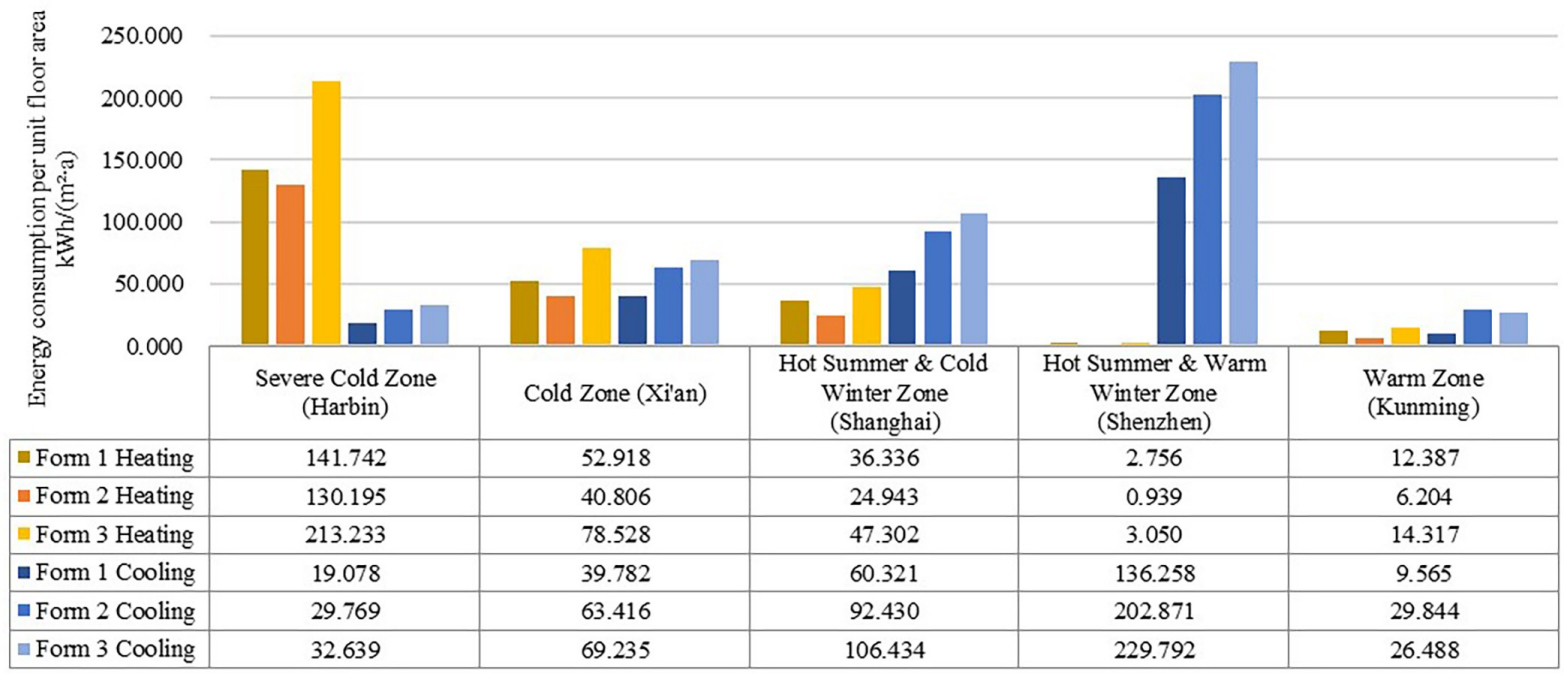
Comparison of simulation results of three typical models in five climate zones.

Heating and cooling energy consumption was simulated considering the three typical patterns in the different climate zones. The results showed (Fig 9) that in terms of heating energy consumption, spatial form 2 exhibited the lowest heating energy consumption, while spatial form 3 attained the highest heating energy consumption. Spatial form 2 achieved a better insulation performance in general and is more suitable for the cold climate zones with lower winter temperatures. In terms of cooling energy consumption, spatial form 3 attained the highest cooling energy consumption, while spatial form 1 reached the lowest cooling energy consumption. Therefore, spatial form 1 is more suitable for the hot climate zones with higher summer temperatures.

#### 3.1.3. Energy-saving rate analysis

**Fig 10:**
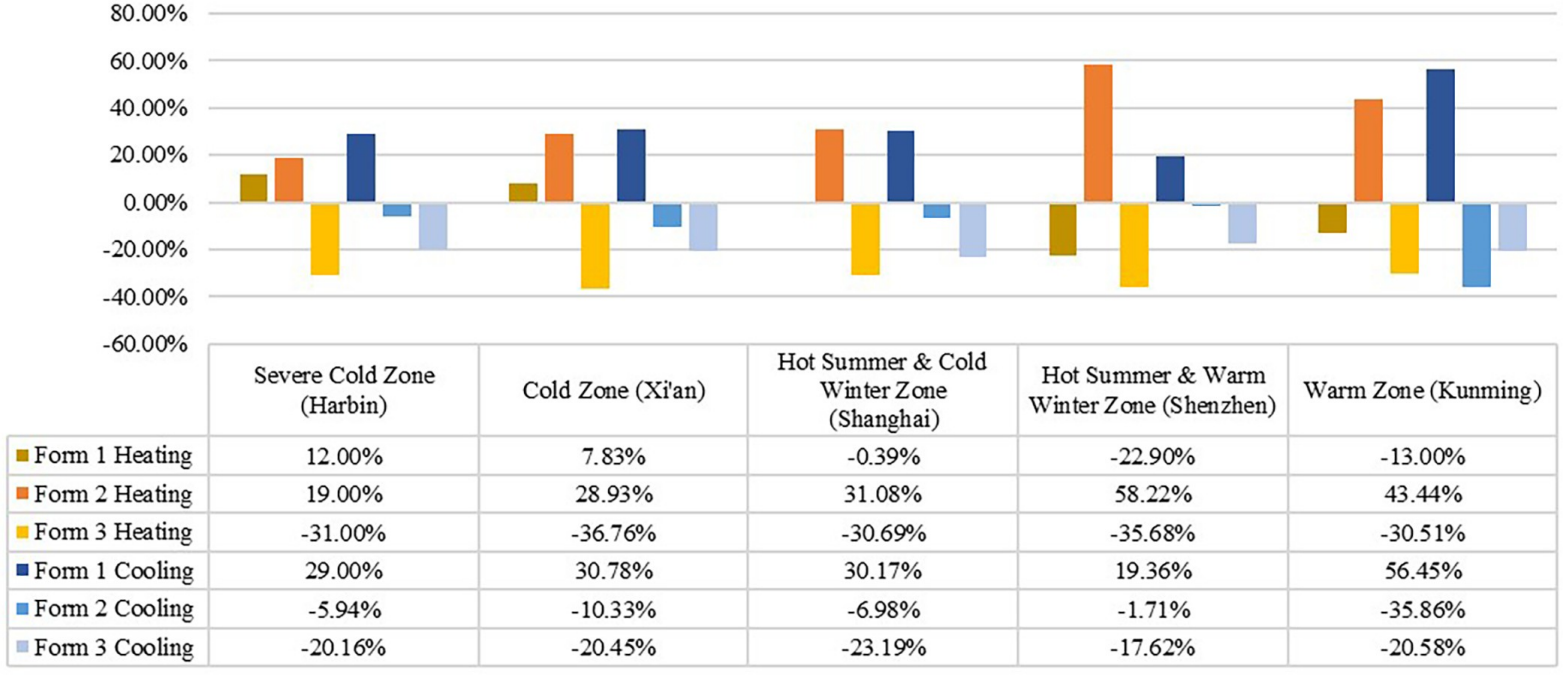
Energy-saving rate between the three typical models in five climate zones.

To further measure the energy-saving effect, the energy-saving rate for heating and cooling energy consumption was calculated for each of the three typical models (Fig 10). The results showed that Form 1 could better reduce the cooling energy consumption, with an energy-saving rate ranging from 19.36%-56.45%, the highest in the warm climate zone. Form 2 could better reduce the heating energy consumption, with an energy-saving rate ranging from 19–58.22%, and this form achieved the highest energy savings in the hot summer and warm winter climate zone. However, Form 3 exhibited a negative energy-saving rate for both heating and cooling, which does not facilitate energy savings. However, the above analysis did not consider possible differences in energy consumption due to building orientation factors.

### 3.2. Analysis of the building orientation effect on energy consumption

The choice of building orientation can also greatly impact energy consumption at the early stages of building design, which is directly related to natural ventilation, lighting, solar radiation, etc. [41]. Based on the survey results of the case orientation in Fig 3, it is clear that outpatient buildings in China are predominantly oriented along the positive direction (i.e., towards the east, south, west, or north) or deviate from the positive direction by a low angle. Therefore, the annual energy consumption was simulated for the three typical models under four main orientations, namely, east, west, south, and north, including the total energy consumption and individual energy consumption (heating and cooling) (Tables 8 and 9). Based on the energy consumption simulation results, the differences in energy consumption due to each building orientation were compared and analysed. Building forms and orientations suitable for the different climate zones were identified to meet the energy-saving requirements and to provide a reference for architects.

**Table 8:** Total energy consumption for the different orientations.

Spatial form	Orientation	Harbin kWh/(m^2^·a)	Xian kWh/(m^2^·a)	Shanghai kWh/m^2^·a)	Shenzhen kWh/(m^2^·a)	Kunming kWh/(m^2^·a)
Form 1	East	*161*.*339*	*96*.*756*	*97*.*279*	139.935	*22*.*019*
	West	161.337	96.482	97.277	*139*.*951*	21.981
	South	160.821	**92.700**	96.657	139.014	21.952
	North	**160.291**	95.653	**96.485**	**139.000**	**21.507**
Form 2	East	161.047	102.863	**117.344**	204.007	**36.035**
	West	160.801	103.456	117.420	204.007	36.109
	South	**159.964**	**103.311**	117.373	**203.811**	36.048
	North	*162*.*500*	*103*.*636*	*117*.*437*	*204*.*040*	*36*.*211*
Form 3	East	*246*.*677*	*148*.*424*	*154*.*349*	*233*.*593*	*41*.*222*
	West	246.503	148.301	154.271	233.475	41.021
	South	245.872	147.763	**153.737**	**232.842**	**40.806**
	North	**245.821**	**147.725**	153.787	232.969	40.941

Note: The data shown in **bold** indicate the lowest energy consumption among the four orientations, and the data shown in *italics* indicate the highest energy consumption.

**Table 9:** Heating and cooling energy consumption for the different orientations.

Spatial form	Orientation	Harbin kWh/(m^2^·a)		Xian kWh/(m^2^·a)		Shanghai kWh/(m^2^·a)		Shenzhen kWh/(m^2^·a)		Kunming kWh/(m^2^·a)	
		Heating	Cooling	Heating	Cooling	Heating	Cooling	Heating	Cooling	Heating	Cooling
Form 1	East	142.032	*19*.*307*	52.895	*43*.*861*	36.324	*60*.*955*	2.732	137.203	12.289	9.730
	West	*142*.*042*	19.295	*53*.*083*	43.399	36.330	60.947	2.724	*137*.*227*	12.270	9.712
	South	141.742	19.078	52.918	**39.782**	*36*.*336*	**60.321**	*2*.*756*	**136.258**	*12*.*387*	*9*.*565*
	North	**141.299**	**18.992**	**52.674**	42.979	**36.160**	60.325	**2.696**	136.304	**12.086**	**9.421**
Form 2	East	131.946	29.100	42.350	**60.513**	24.941	92.403	0.931	203.076	6.156	29.878
	West	131.164	29.636	**41.777**	*61*.*679*	**24.925**	*92*.*495*	**0.929**	203.078	**6.151**	*29*.*958*
	South	**130.195**	*29*.*769*	41.785	61.526	24.943	92.430	*0*.*939*	**202.871**	6.204	**29.844**
	North	*133*.*497*	**29.003**	*42*.*648*	60.988	*25*.*177*	**92.260**	0.936	*203*.*104*	*6*.*320*	29.892
Form 3	East	*213*.*696*	*32*.*980*	*78*.*692*	*69*.*732*	*47*.*352*	*106*.*997*	*3*.*062*	*230*.*531*	*14*.*400*	*26*.*822*
	West	213.658	32.845	78.629	69.673	47.324	106.947	**3.044**	230.432	**14.298**	26.723
	South	**213.233**	**32.639**	**78.528**	69.235	**47.302**	**106.434**	3.050	**229.792**	14.317	**26.488**
	North	213.235	32.586	78.546	**69.179**	47.335	106.452	3.058	229.911	14.385	26.556

Note: The data shown in **bold** indicate the lowest energy consumption among the four orientations, and the data shown in *italics* indicate the highest energy consumption.

#### 3.2.1. Detailed discussion of the results for the severe cold zone (Harbin)

The total energy consumption results in Table 8 show that there was less variation in the results of the same typical model within the same climate zone but a higher variation in the results of the different typical models. This indicates that the building form significantly influenced the simulation results. In the severe cold climate zone, Form 2 (southwards) yielded the lowest total energy consumption, at 159.964 kWh/(m^2^·a), while Form 3 (northwards) yielded the highest total energy consumption, at 245.821 kWh/(m^2^·a). Hence, Form 2 (southwards) is more suitable for harsher climate zones. The individual energy consumption results in Table 8 show that the energy for heating was much higher than that for cooling in cold climates, and therefore, building forms with low heating energy consumption should be considered first. Form 2 (southwards) attained the lowest heating energy consumption, at 130.195 kWh/(m^2^·a), while Form 3 (southwards) attained the highest heating energy consumption, at 213.233 kWh/(m^2^·a). There was a slight difference in cooling energy consumption between the three typical models, with the cooling energy consumption ranked as Form 3 > Form 2 > Form 1.

**Fig 11:**
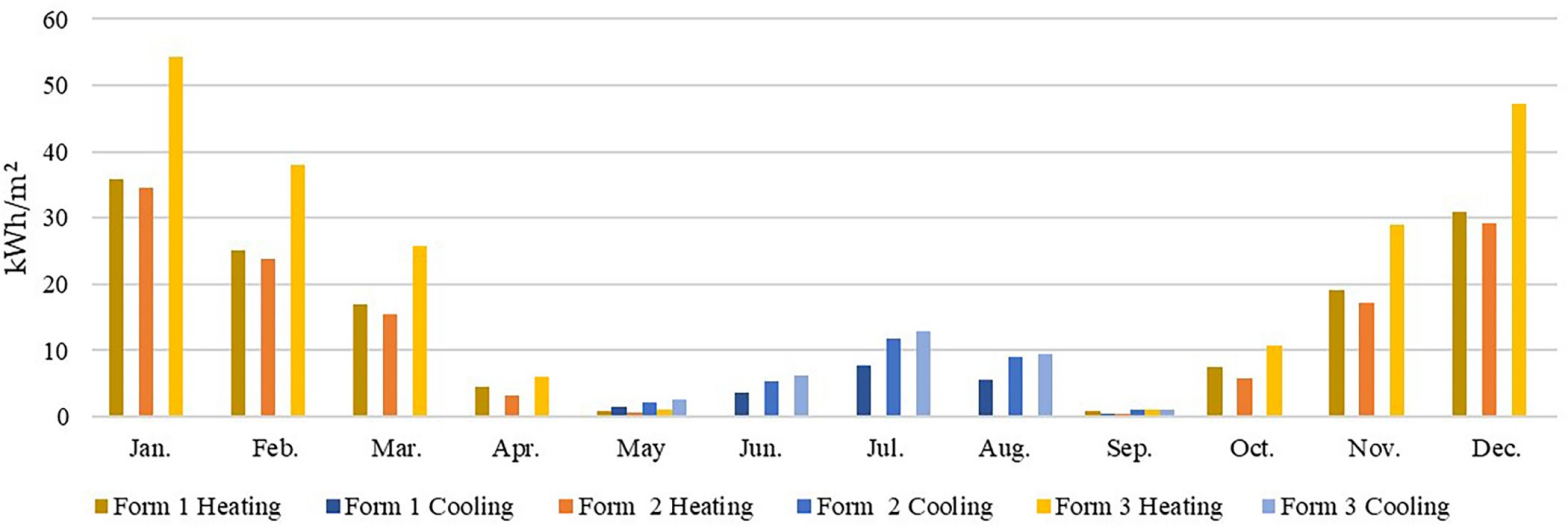
Monthly heating and cooling energy consumption in Harbin.

In the cold climate zone, Harbin, for example, the heating energy consumption was much higher than the cooling energy consumption throughout the year (Fig 11). Therefore, we should prioritize building forms and orientations that reduce the heating energy consumption so that the long side of the building receives sufficient solar radiation. In summary, the choice of building form 2 (southwards) better facilitates energy efficiency improvement.

#### 3.2.2. Detailed discussion of the results for the cold zone (Xi’an)

In the cold climate zone, as indicated by the total energy consumption results in Table 8, Form 1 (southwards) yielded the lowest energy consumption, at 92.700 kWh/(m^2^·a), while Form 3 (northwards) yielded the highest energy consumption, at 147.725 kWh/(m^2^·a). Therefore, Form 1 (southwards) is more suitable for the cold climate zone. The individual energy consumption results in Table 8 show that the heating and cooling energy consumption values were more evenly distributed and should be jointly considered. Regarding heating energy consumption, Form 2 (westwards) attained the lowest energy consumption, at 41.777 kWh/(m^2^·a), while Form 3 (southwards) attained the highest energy consumption, at 78.528 kWh/(m^2^·a). In terms of cooling energy consumption, Form 1 (southwards) achieved the lowest value, at 39.782 kWh/(m^2^·a), while Form 3 (northwards) achieved the highest value, at 69.179 kWh/(m^2^·a).

**Fig 12:**
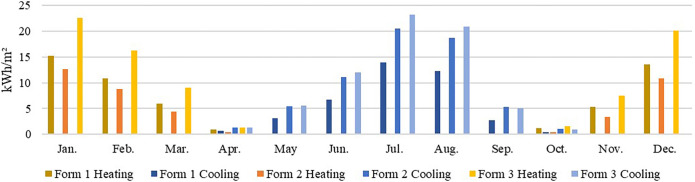
Monthly heating and cooling energy consumption in Xi’an.

In the cold climate zone represented by Xi’an, the building design should account for both the winter heating and summer cooling requirements (Fig 12). Therefore, of the three forms and the different orientations, the one with the lowest heating and cooling energy consumption is preferred. Based on the simulation results, Form 1 (southwards) should be chosen, which exhibited the lowest total and cooling energy consumption but a slightly higher heating energy consumption in winter. Hence, it is necessary to consider strengthening insulation measures in winter.

#### 3.2.3. Detailed discussion of the results for the hot summer and cold winter zone (Shanghai)

In the hot summer and cold winter climate zone, the total energy consumption results in Table 8 show that Form 1 (northwards) generated the lowest energy consumption, at 96.485 kWh/(m^2^·a), while Form 3 (southwards) generated the highest energy consumption, at 153.737 kWh/(m^2^·a). Therefore, Form 1 (northwards) is more suitable for the hot summer and cold winter zone. The individual energy consumption results in Table 8 show that the cooling energy consumption was approximately 2–3 times higher than the heating energy consumption, at approximately 60–106 kWh/(m^2^·a) and 24–47 kWh/(m^2^·a), respectively. Therefore, in this climate zone, cooling energy consumption should be primarily considered. In terms of cooling energy consumption, Form 1 (northwards) attained the lowest energy consumption, at 96.485 kWh/(m^2^·a), while Form 3 (southwards) attained the highest energy consumption, at 153.737 kWh/(m^2^·a). Regarding heating energy consumption, Form 2 (westwards) yielded the lowest energy consumption, at 24.925 kWh/(m^2^·a), while Form 3 (southwards) yielded the highest energy consumption, at 47.302 kWh/(m^2^·a).

**Fig 13:**
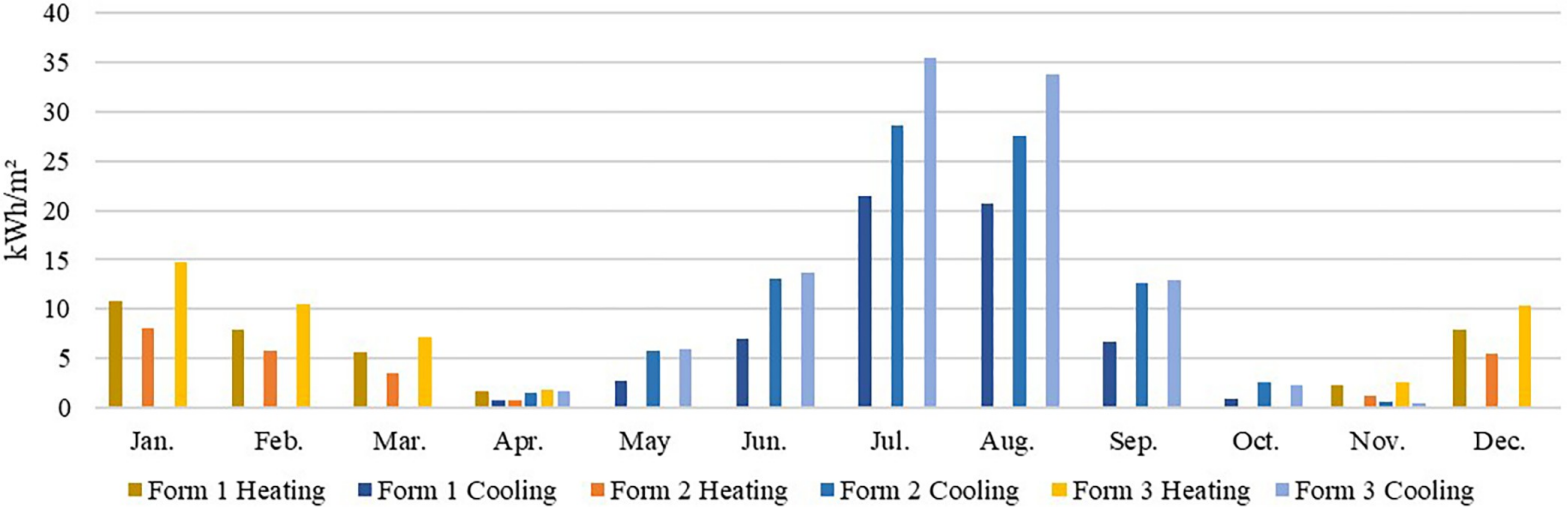
Monthly heating and cooling energy consumption in Shanghai.

In the hot summer and cold winter climate zone, adopting Shanghai as an example, based on the year-round energy consumption distribution (Fig 13), the summer cooling energy consumption should mainly be considered in building form selection. The one with the lowest total energy consumption and cooling energy consumption should be chosen. Therefore, based on the above analysis, a building of Form 1 facing south should be chosen to achieve a higher energy efficiency.

#### 3.2.4. Detailed discussion of the results for the hot summer and warm winter zone (Shenzhen)

In the hot summer and cold winter climate zone, the total energy consumption of the three typical models ranged from 139~232.842 kWh/(m^2^·a), with a high potential for energy savings. The lowest total energy consumption was found in model 1 (northwards), and the highest was observed in model 3 (southwards). Table 8 shows that the climate zone was dominated by cooling energy consumption, with negligible heating energy consumption. Therefore, only forms with a low cooling energy consumption should be considered. (2) The cooling energy consumption under Form 1 slightly differed between the various directions, with Form 1 (southwards) attaining the lowest energy consumption, at 136.258 kWh/(m^2^ -a). Form 3 (southwards) exhibited the highest energy consumption, at 229.792 kWh/(m^2^·a).

**Fig 14:**
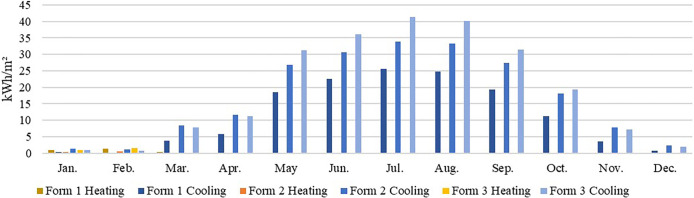
Monthly heating and cooling energy consumption in Shenzhen.

In the hot summer and winter climate zone, e.g., in Shenzhen, the heating energy consumption of all 3 forms under all orientations was low, mainly because the region exhibits high temperatures for most of the year (Fig 14) (average temperature >10°C in January and 25°C- 29°C in July), dominated by the cooling demand. The main consideration in building form design is cooling energy consumption reduction. Therefore, in this climate zone, Form 1 facing south should be chosen.

#### 3.2.5. Detailed discussion of the results for the warm zone (Kunming)

In the warm climate zone, the results in Table 9 show that the total energy consumption was low for all three typical models, with energy consumption values ranging from 21.507~40.806 kWh/(m^2^·a), with a negligible difference. This indicates that changes in the building orientation in this area do not significantly impact energy consumption. Of the three typical models, Form 1 (northwards) attained the lowest total energy consumption, while Form 3 (southwards) attained the highest total energy consumption. The results in Table 8 show that the overall difference between the cooling and heating energy consumption values of the three typical models in this climate zone was not significant, and all models yielded low values. In terms of heating energy consumption, Form 2 (westwards) yielded the lowest energy consumption, at 6.151 kWh/(m^2^·a), while Form 3 (westwards) attained the highest energy consumption, at 14.298 kWh/(m^2^·a). In terms of cooling energy consumption, Form 1 (northwards) achieved the lowest energy consumption, at 9.421 kWh/(m^2^·a), while Form 2 (southwards) achieved the highest energy consumption, at 29.844 kWh/(m^2^·a).

**Fig 15:**
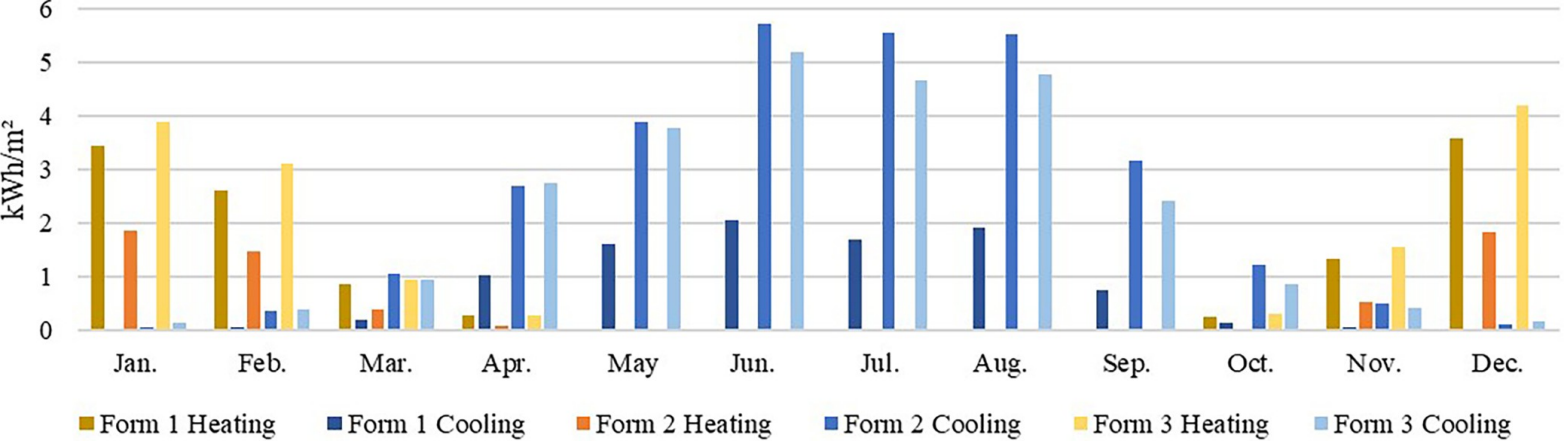
Monthly heating and cooling energy consumption in Kunming.

In the warm climate zone, adopting Kunming as an example, where the overall demand for heating and cooling is low due to the suitable annual average temperatures (0–13°C in January and 18–25°C in July), the impacts of the building form and orientation on energy consumption are minimal (Fig 15). Therefore, Forms 1 and 2 with a low total energy consumption should be selected at the early stages of building design.

## 4. Discussion

Energy performance assessment of building forms is important for architects and engineers to make decisions about achieving high-performance buildings or low-energy buildings. This study analyses the heating and cooling energy consumption of three typical outpatient building forms under five climatic conditions from the perspective of the early stages of building design. In addition, the energy consumption of each form under different orientations is compared.

In terms of the effect of building form on energy consumption, the energy consumption of the three spatial forms shows the same trend across the five climate zones, Form 3 > Form 2 > Form 1, indicating that the outpatient building form has a significant effect on energy consumption. Comparing the simulation results for the five climate zones, the total energy consumption is lowest in the warm climate zone, and relatively high in the cold climate zone and the hot summer/cold winter climate zone, with the latter being about 4–8 times higher than the former. This result indicates that heating and cooling energy consumption accounts for a large proportion of building energy consumption, which is consistent with the findings of [42, 43]. In terms of the effect of building orientation on energy consumption, in general, the energy consumption of outpatient buildings with different orientations does not vary much across climatic zones, mainly due to the symmetrical layout of the clinic space, which is generally unitary, with similar window layouts on all facades except the main entrance. Compared with [12, 13], this study expands the spatial scope to include five climate zones in China and identifies appropriate building forms for each climate zone. The factors affecting building energy consumption were simplified, and building form and orientation were studied.

It is important to note that most buildings remain unique and their trends will change over time. Nevertheless, the results of these studies can provide architects and engineers with general information about the energy-efficient design of outpatient buildings, enabling them to predict the energy consumption of a building simply and quickly at the beginning of the planning phase. Our findings suggest that even in general, there are rules that architects can follow when designing spatial forms to adjust the shape and orientation of buildings to adapt to the climate and achieve the highest level of energy efficiency. Reducing carbon emissions in the context of global pandemics and rapid urban expansion.

However, there are certain limitations to this study. For example, the impact of the surrounding environment was not considered when determining the energy consumption of these models. The building’s surroundings influence solar radiation, natural lighting, and ventilation. Considering these factors, the building forms and orientations that facilitate energy efficiency improvement could differ from the study results. In addition, only three typical patterns of outpatient buildings in China were analysed in this study. As the spatial parameters are continuously optimized, the energy consumption of other typical models should be studied in the future and analysed in greater detail to ensure the sustainable energy-efficient design of outpatient buildings.

## 5. Conclusion

Optimization of the spatial form and orientation of outpatient buildings can significantly reduce energy consumption, and differences in the form of the door building have a greater impact on the building’s operational energy consumption than changes in the building’s orientation, all other parameters being equal. In the five climatic zones, the total energy consumption values showed the same trend from low to high, centralised (21.952–160.821 kWh/(m^2^∙a)), connecting corridor (36.048–203.811 kWh/(m^2^∙a)) and courtyard (40.806–245.872 kWh/(m^2^∙a)).

Considering both the building form and orientation, the following recommendations for building selection in the five climate zones were made: Form 2 (southwards) should be selected in the severely cold zone; Form 1 (southwards) should be selected in the cold zone and the hot summer and cold winter zone; Form 1 (northwards) should be selected in the hot summer and warm winter zone; and Form 1 or 2 can be selected in the warm zone. However, among the three typical forms, the courtyard form (Form 3) attained the highest energy consumption, which is not conducive to energy efficiency enhancement. If this building form is chosen, measures should be implemented in other areas to reduce energy consumption.

## Supporting information

S1 Fig Typical forms of outpatient buildings and their energy consumption. (PDF)

S1 Table Energy consumption of three building forms in five climate zones. (XLSX)
