# Supplementary material for: Effect of the spatial form of outpatient buildings on energy consumption in different climate zones in China
Source: PLoS One. 2023 Nov 9;18(11):e0293982. doi: 10.1371/journal.pone.0293982 (PMC10635504; doi:10.1371/journal.pone.0293982)
Supplement: S1 Fig — (PDF) [file pone.0293982.s001.pdf]

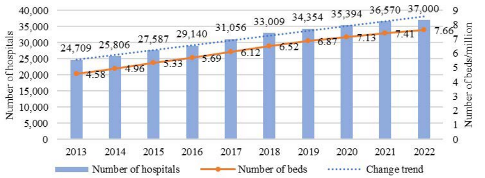
40,000
35,000
30,000
25,000
20,000
15,000
10,000
5,000
0
Number of hospitals
9
8
7
6
5
4
3
2
1
0
Number of beds/million
24,709
25,806
27,587
29,140
31,056
33,009
34,354
35,394
36,570
37,000
4.58
4.96
5.33
5.69
6.12
6.52
6.87
7.13
7.41
7.66
2013
2014
2015
2016
2017
2018
2019
2020
2021
2022
Number of hospitals
Number of beds
Change trend

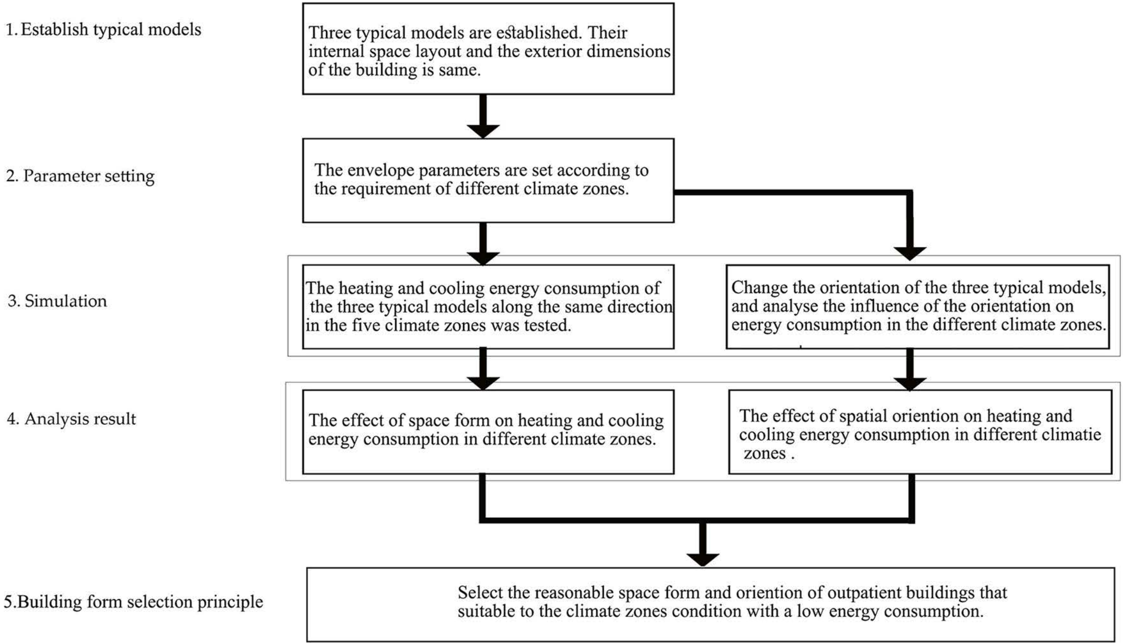
1. Establish typical models
Three typical models are established. Their internal space layout and the exterior dimensions of the building is same.
2. Parameter setting
The envelope parameters are set according to the requirement of different climate zones.
3. Simulation
The heating and cooling energy consumption of the three typical models along the same direction in the five climate zones was tested.
Change the orientation of the three typical models, and analyse the influence of the orientation on energy consumption in the different climate zones.
4. Analysis result
The effect of space form on heating and cooling energy consumption in different climate zones.
The effect of spatial oriention on heating and cooling energy consumption in different climatie zones .
5.Building form selection principle
Select the reasonable space form and oriention of outpatient buildings that suitable to the climate zones condition with a low energy consumption.

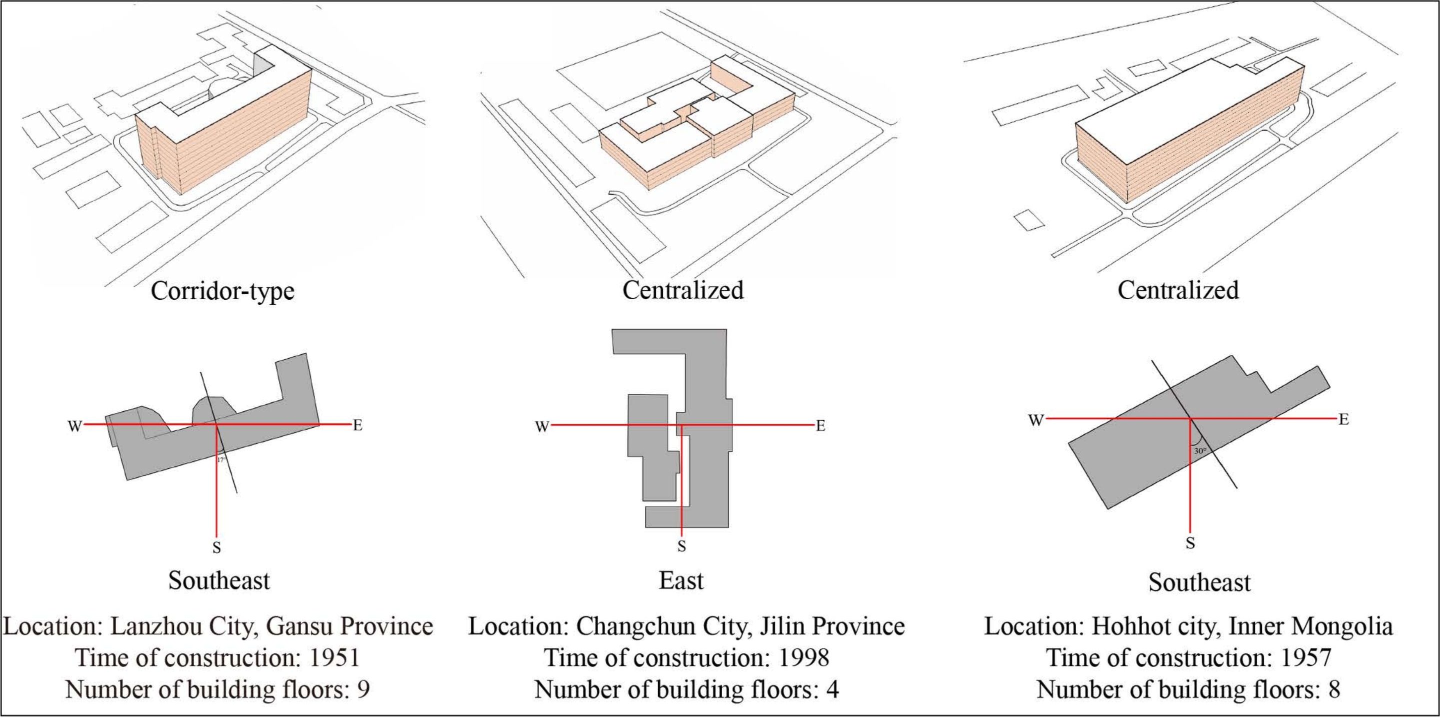
Corridor-type
Centralized
Centralized
W
E
S
Southeast
W
E
S
East
W
E
30°
S
Southeast
Location: Lanzhou City, Gansu Province
Time of construction: 1951
Number of building floors: 9
Location: Changchun City, Jilin Province
Time of construction: 1998
Number of building floors: 4
Location: Hohhot city, Inner Mongolia
Time of construction: 1957
Number of building floors: 8

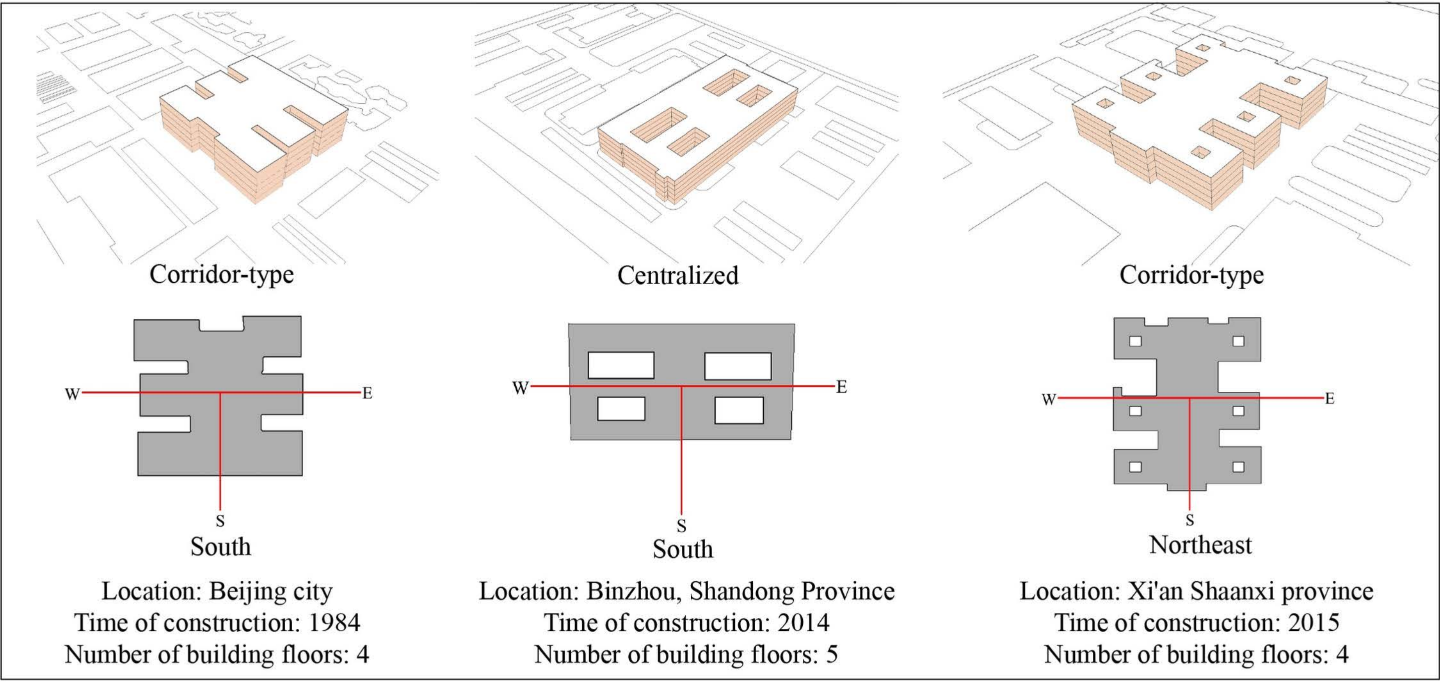
Corridor-type
W
E
S
South
Location: Beijing city
Time of construction: 1984
Number of building floors: 4
Centralized
W
E
S
South
Location: Binzhou, Shandong Province
Time of construction: 2014
Number of building floors: 5
Corridor-type
W
E
S
Northeast
Location: Xi'an Shaanxi province
Time of construction: 2015
Number of building floors: 4

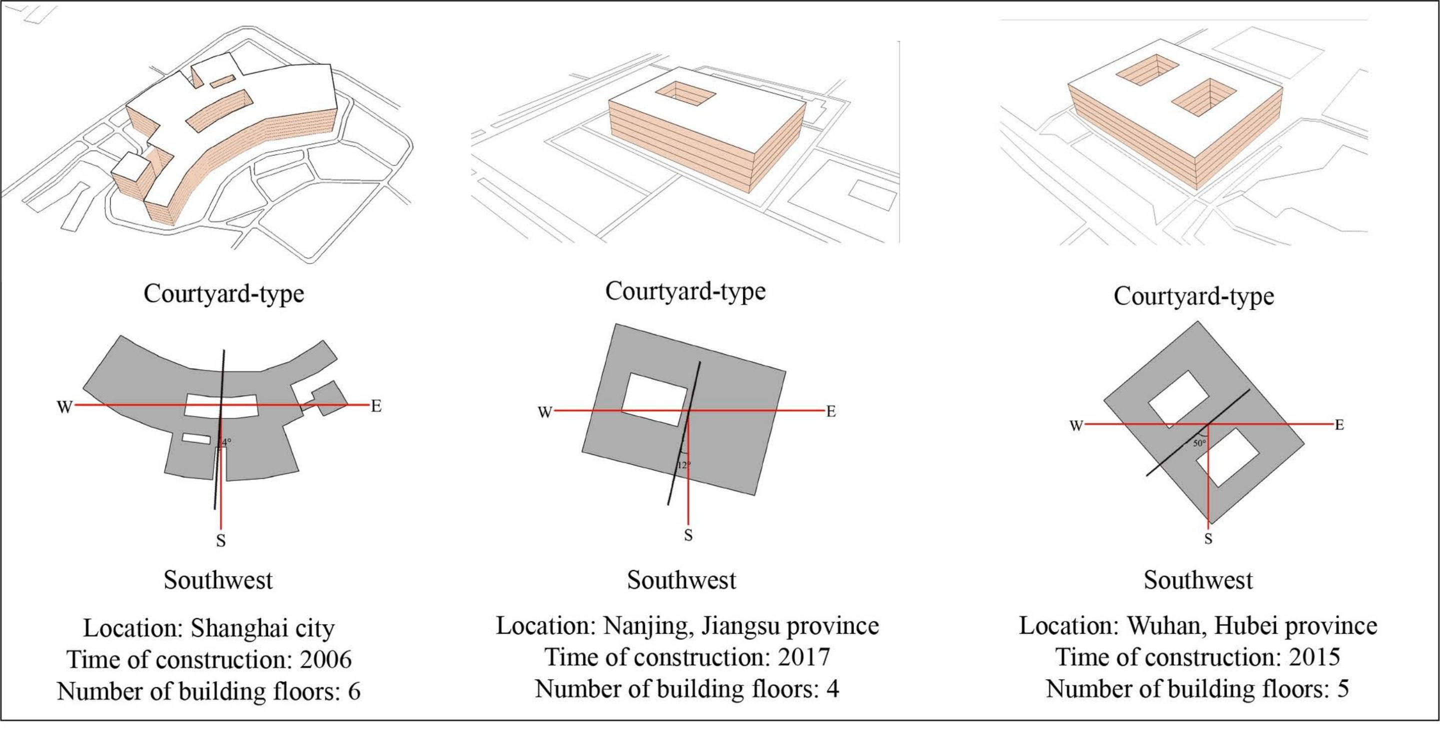

Courtyard-type
W
E
S
4°
Southwest
Location: Shanghai city
Time of construction: 2006
Number of building floors: 6
Courtyard-type
W
E
S
12°
Southwest
Location: Nanjing, Jiangsu province
Time of construction: 2017
Number of building floors: 4
Courtyard-type
W
E
S
50°
Southwest
Location: Wuhan, Hubei province
Time of construction: 2015
Number of building floors: 5

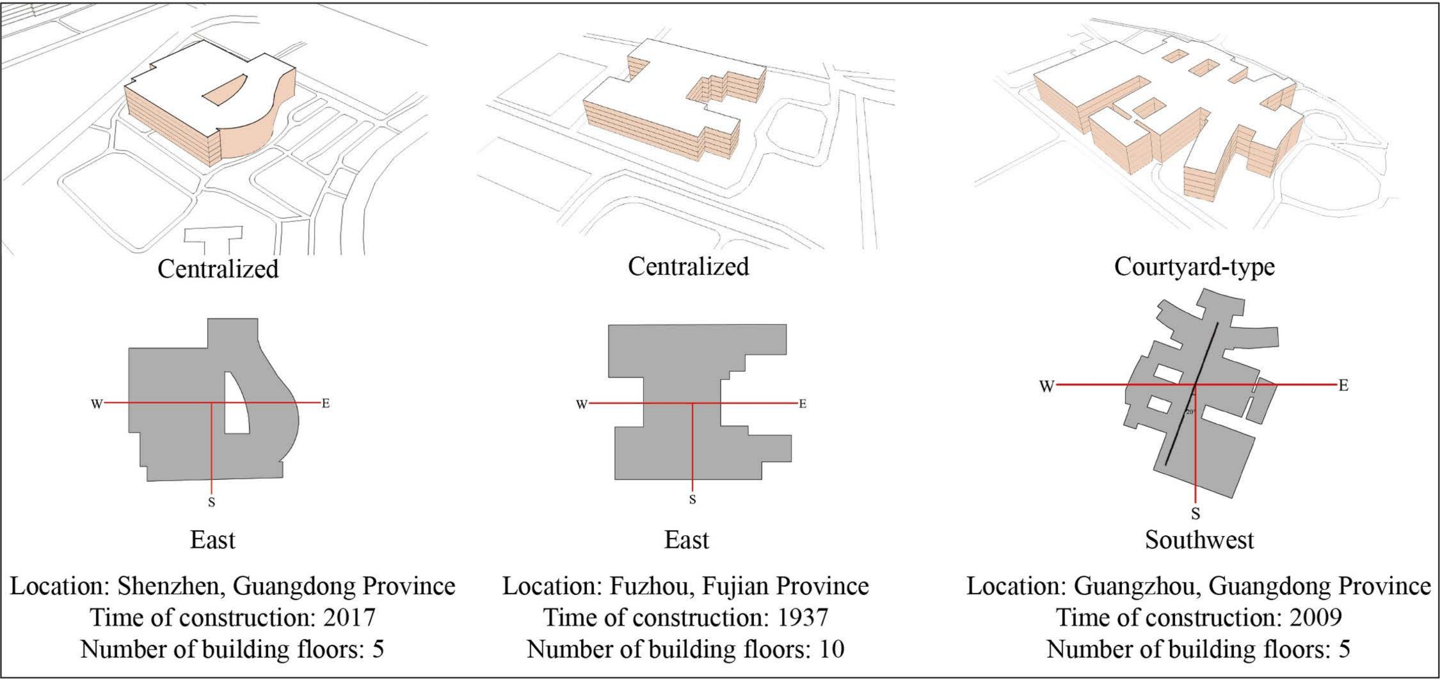
Centralized
Centralized
Courtyard-type
W
E
S
W
E
S
W
E
S
East
East
Southwest
Location: Shenzhen, Guangdong Province
Time of construction: 2017
Number of building floors: 5
Location: Fuzhou, Fujian Province
Time of construction: 1937
Number of building floors: 10
Location: Guangzhou, Guangdong Province
Time of construction: 2009
Number of building floors: 5

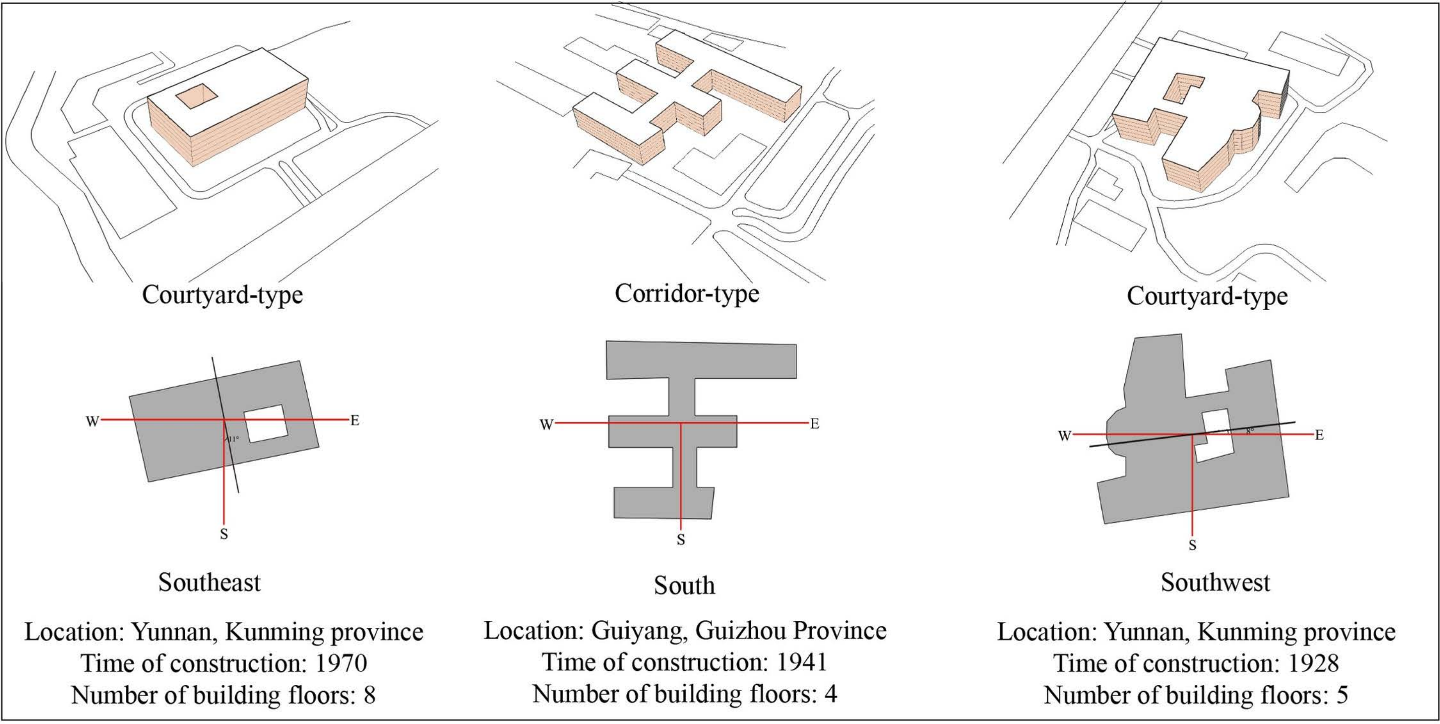
Courtyard-type
Corridor-type
Courtyard-type
W
E
S
11°
W
E
S
W
E
S
8°
Southeast
South
Southwest
Location: Yunnan, Kunming province
Time of construction: 1970
Number of building floors: 8
Location: Guiyang, Guizhou Province
Time of construction: 1941
Number of building floors: 4
Location: Yunnan, Kunming province
Time of construction: 1928
Number of building floors: 5

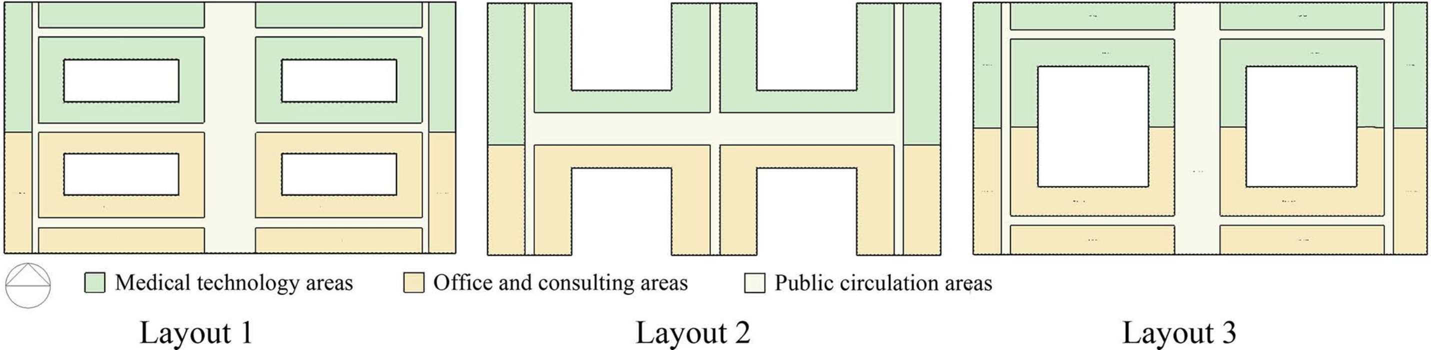

Medical technology areas
Office and consulting areas
Public circulation areas
Layout 1
Layout 2
Layout 3

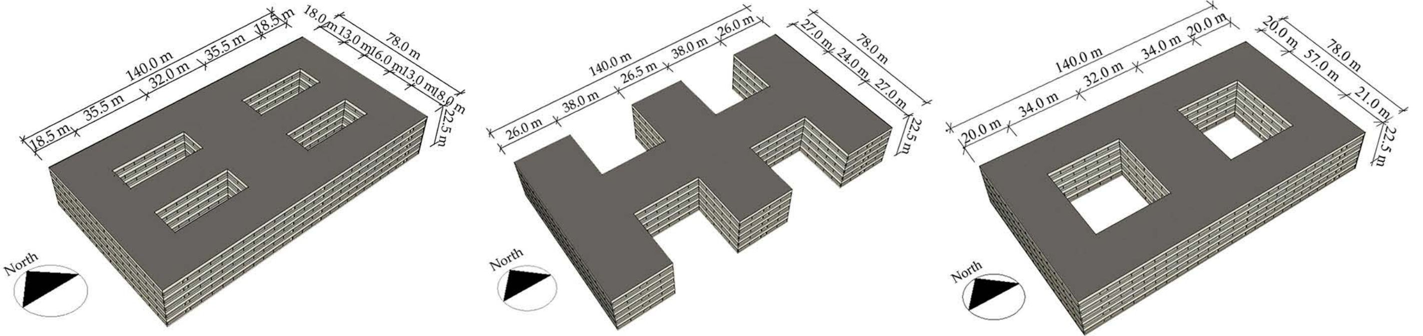
140.0 m
18.5 m
35.5 m
32.0 m
35.5 m
18.5 m
78.0 m
18.0 m
13.0 m
16.0 m
13.0 m
18.0 m
22.5 m
North
140.0 m
26.0 m
38.0 m
26.5 m
38.0 m
26.0 m
78.0 m
27.0 m
24.0 m
27.0 m
22.5 m
North
140.0 m
20.0 m
34.0 m
32.0 m
34.0 m
20.0 m
78.0 m
20.0 m
57.0 m
21.0 m
22.5 m
North

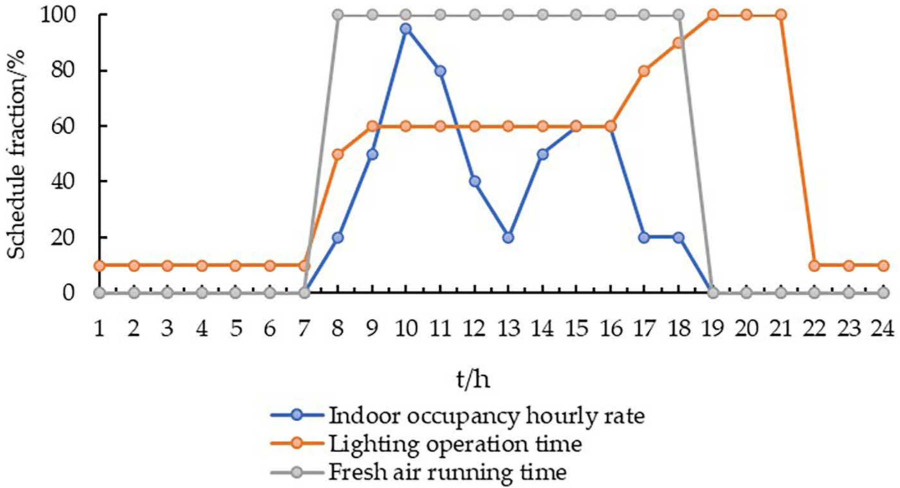

Schedule fraction/%
100
80
60
40
20
0
1 2 3 4 5 6 7 8 9 10 11 12 13 14 15 16 17 18 19 20 21 22 23 24
t/h
Indoor occupancy hourly rate
Lighting operation time
Fresh air running time

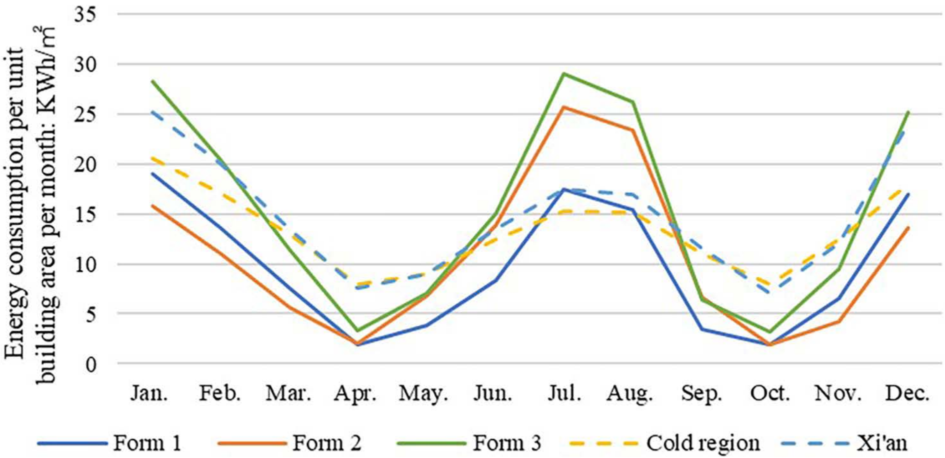

Energy consumption per unit building area per month: KWh/m²
0
5
10
15
20
25
30
35
Jan.
Feb.
Mar.
Apr.
May.
Jun.
Jul.
Aug.
Sep.
Oct.
Nov.
Dec.
Form 1
Form 2
Form 3
Cold region
Xi'an

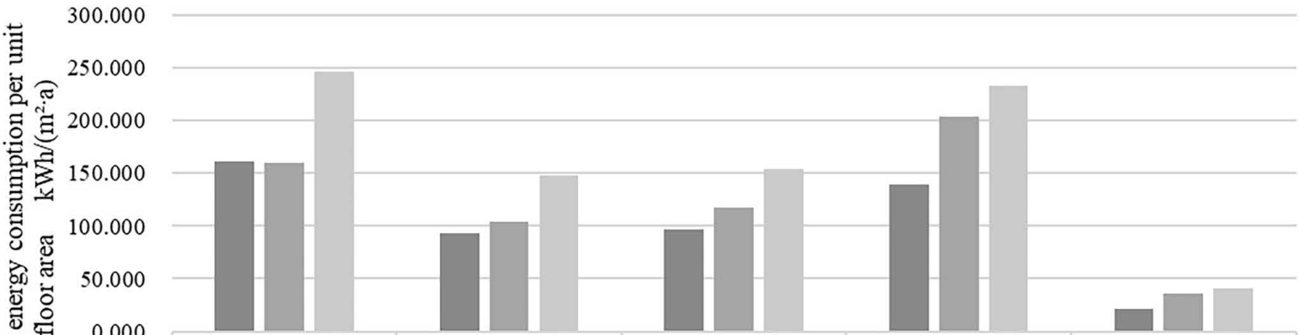

	Severe Cold Zone (Harbin)	Cold Zone (Xi'an)	Hot Summer & Cold Winter Zone (Shanghai)	Hot Summer & Warm Winter Zone (Shenzhen)	Warm Zone (Kunming)
Form 1	160.821	92.700	96.657	139.014	21.952
Form 2	159.964	104.223	117.373	203.811	36.048
Form 3	245.872	147.763	153.737	232.842	40.806

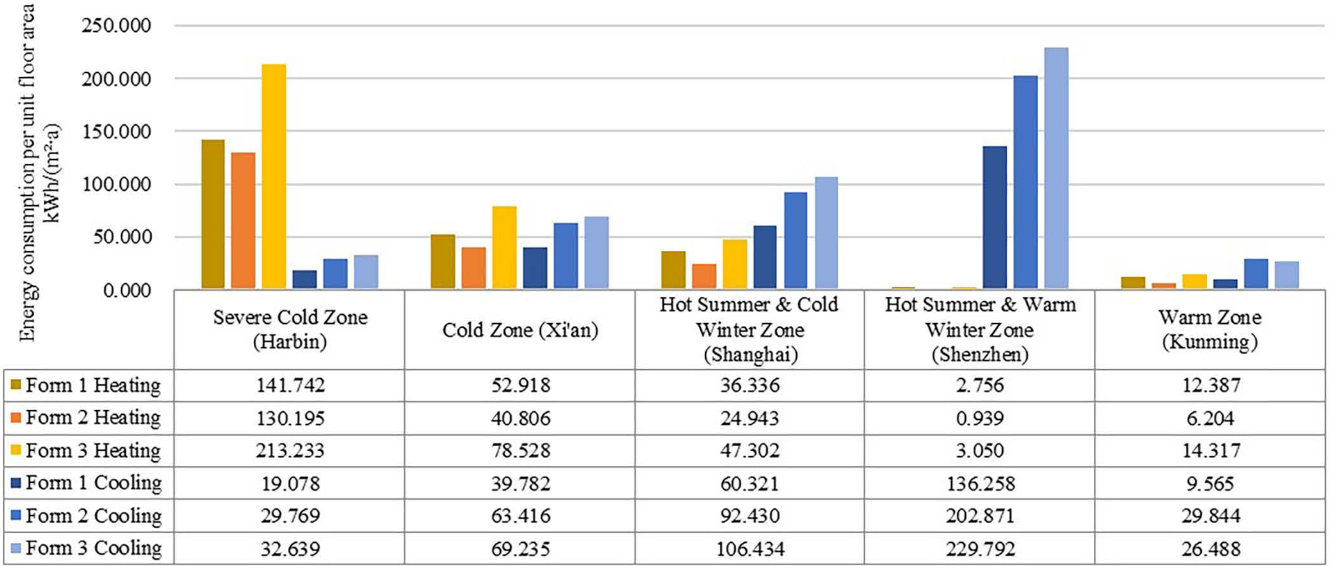

	Severe Cold Zone (Harbin)	Cold Zone (Xi'an)	Hot Summer & Cold Winter Zone (Shanghai)	Hot Summer & Warm Winter Zone (Shenzhen)	Warm Zone (Kunming)
Form 1 Heating	141.742	52.918	36.336	2.756	12.387
Form 2 Heating	130.195	40.806	24.943	0.939	6.204
Form 3 Heating	213.233	78.528	47.302	3.050	14.317
Form 1 Cooling	19.078	39.782	60.321	136.258	9.565
Form 2 Cooling	29.769	63.416	92.430	202.871	29.844
Form 3 Cooling	32.639	69.235	106.434	229.792	26.488

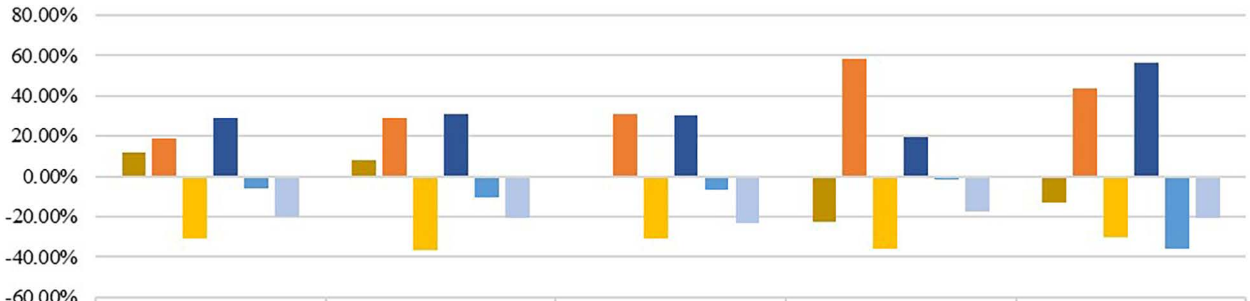

	Severe Cold Zone (Harbin)	Cold Zone (Xi'an)	Hot Summer & Cold Winter Zone (Shanghai)	Hot Summer & Warm Winter Zone (Shenzhen)	Warm Zone (Kunming)
Form 1 Heating	12.00%	7.83%	-0.39%	-22.90%	-13.00%
Form 2 Heating	19.00%	28.93%	31.08%	58.22%	43.44%
Form 3 Heating	-31.00%	-36.76%	-30.69%	-35.68%	-30.51%
Form 1 Cooling	29.00%	30.78%	30.17%	19.36%	56.45%
Form 2 Cooling	-5.94%	-10.33%	-6.98%	-1.71%	-35.86%
Form 3 Cooling	-20.16%	-20.45%	-23.19%	-17.62%	-20.58%

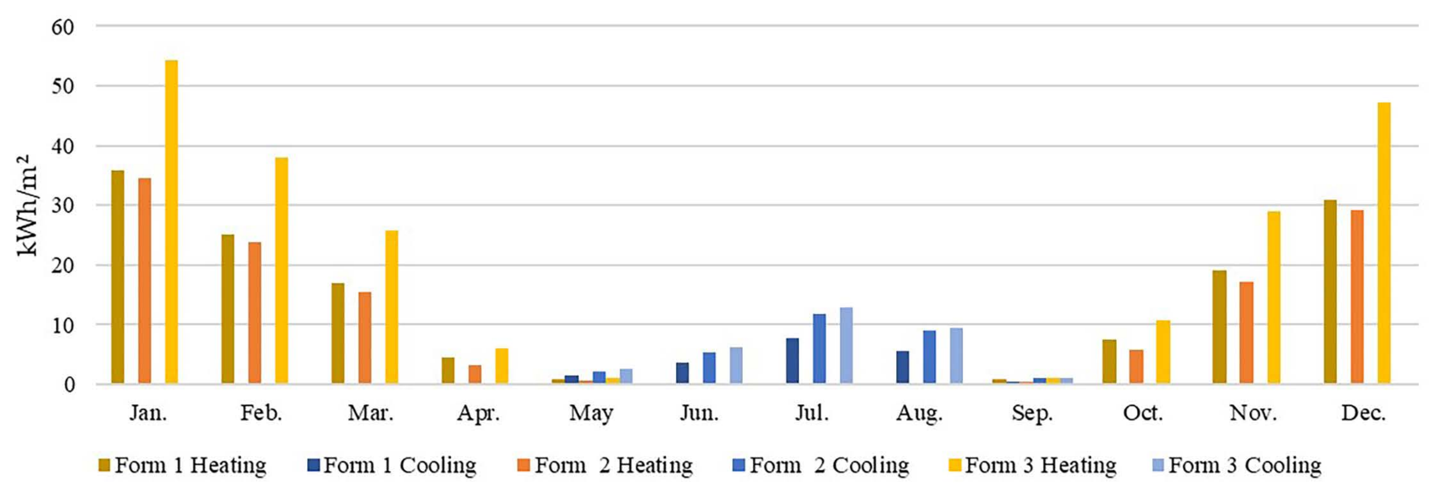

60
50
40
30
20
10
0
kWh/m²
Jan.
Feb.
Mar.
Apr.
May
Jun.
Jul.
Aug.
Sep.
Oct.
Nov.
Dec.
Form 1 Heating
Form 1 Cooling
Form 2 Heating
Form 2 Cooling
Form 3 Heating
Form 3 Cooling

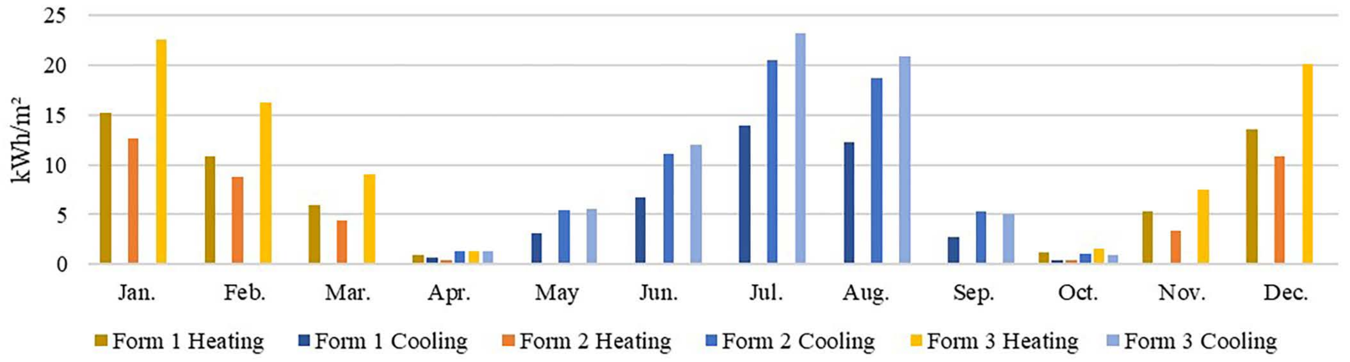
kWh/m²
25
20
15
10
5
0
Jan.
Feb.
Mar.
Apr.
May
Jun.
Jul.
Aug.
Sep.
Oct.
Nov.
Dec.
Form 1 Heating
Form 1 Cooling
Form 2 Heating
Form 2 Cooling
Form 3 Heating
Form 3 Cooling

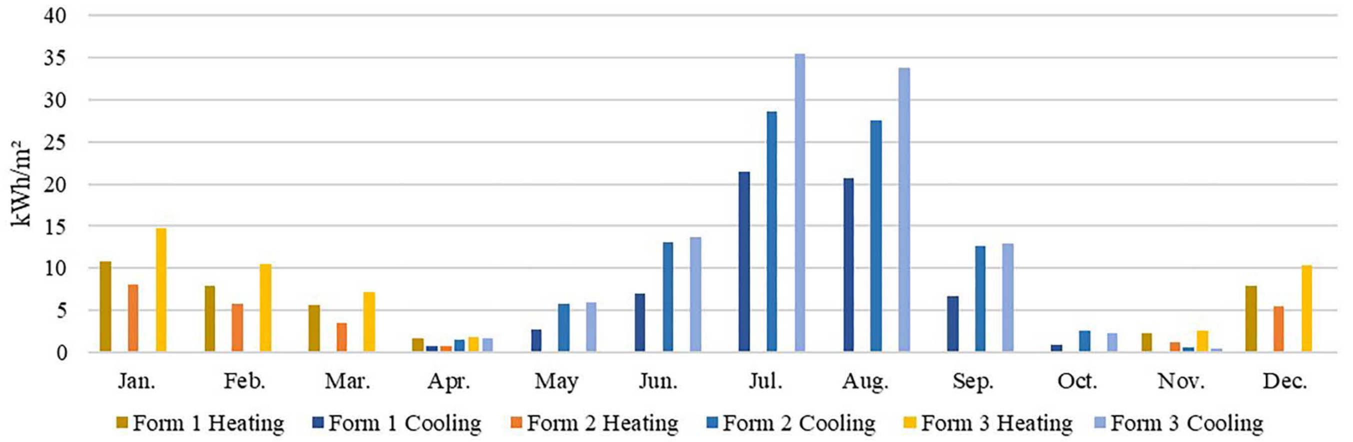
kWh/m²
40
35
30
25
20
15
10
5
0
Jan.
Feb.
Mar.
Apr.
May
Jun.
Jul.
Aug.
Sep.
Oct.
Nov.
Dec.
Form 1 Heating
Form 1 Cooling
Form 2 Heating
Form 2 Cooling
Form 3 Heating
Form 3 Cooling

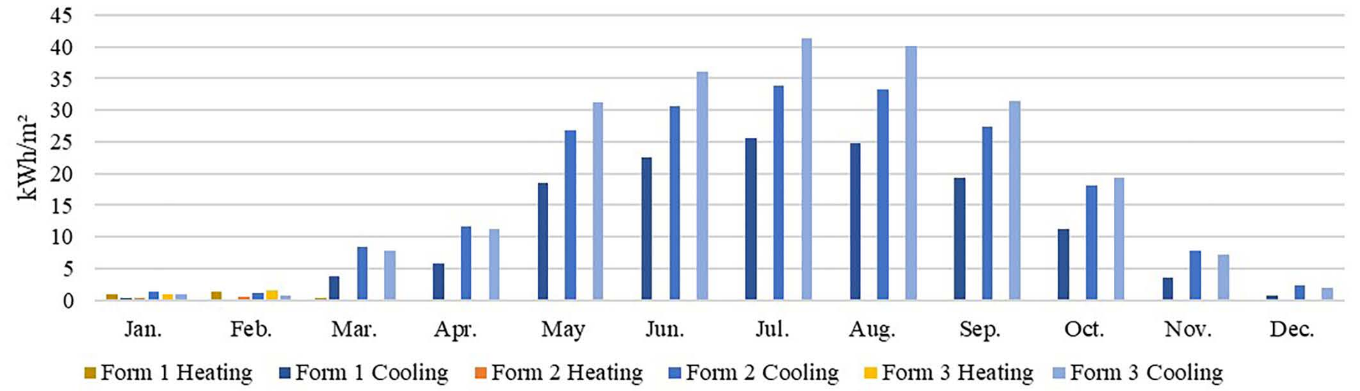
45
40
35
30
25
20
15
10
5
0
kWh/m²
Jan.
Feb.
Mar.
Apr.
May
Jun.
Jul.
Aug.
Sep.
Oct.
Nov.
Dec.
Form 1 Heating
Form 1 Cooling
Form 2 Heating
Form 2 Cooling
Form 3 Heating
Form 3 Cooling

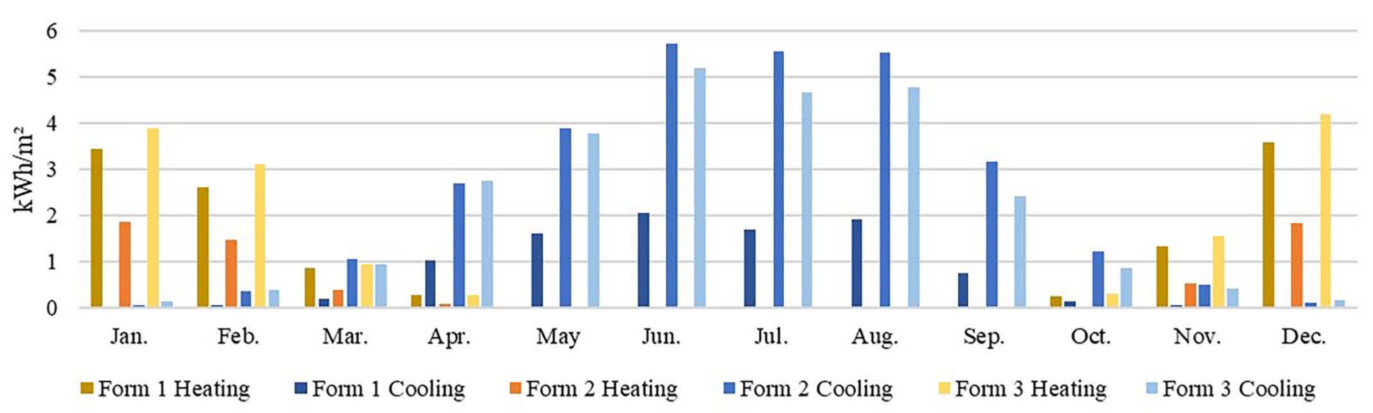

6
5
4
3
2
1
0
kWh/m²
Jan.
Feb.
Mar.
Apr.
May
Jun.
Jul.
Aug.
Sep.
Oct.
Nov.
Dec.
Form 1 Heating
Form 1 Cooling
Form 2 Heating
Form 2 Cooling
Form 3 Heating
Form 3 Cooling
